# Genomic diversity of *Salmonella enterica* serovar Typhimurium isolated from chicken processing facilities in New South Wales, Australia

**DOI:** 10.3389/fmicb.2024.1440777

**Published:** 2024-08-14

**Authors:** Samitha Bandaranayake, Sarah Williamson, Jack Stewart, Michael Payne, Sandeep Kaur, Qinning Wang, Vitali Sintchenko, Anthony Pavic, Ruiting Lan

**Affiliations:** ^1^School of Biotechnology and Biomolecular Sciences, University of New South Wales, Sydney, NSW, Australia; ^2^Birling Laboratories, Bringelly, NSW, Australia; ^3^Centre for Infectious Diseases and Microbiology-Laboratory Services, Institute of Clinical Pathology and Medical Research, NSW Health Pathology, Westmead Hospital, Westmead, NSW, Australia; ^4^Marie Bashir Institute for Infectious Diseases and Biosecurity, Sydney Medical School, University of Sydney, Sydney, NSW, Australia

**Keywords:** *Salmonella* Typhimurium, sequence type, multilevel genome typing, meat chicken, carcass

## Abstract

Contamination of poultry products by *Salmonella enterica* serovar Typhimurium (STm) is a major cause of foodborne infections and outbreaks. This study aimed to assess the diversity and antimicrobial resistance (AMR) carriage of STm in three chicken processing plants using genomic sequencing. It also aimed to investigate whether any particular strain types were associated with cases of human illness. Multilevel genome typing (MGT) was used to analyze 379 STm isolates from processed chicken carcasses. The diversity of chicken STm sequence types (STs) increased from MGT1 (2 STs) to MGT9 (257 STs). STs at MGT5 to MGT9 levels that were unique to one processing plant and shared among the processing plants were identified, likely reflecting the diversity of STm at their farm source. Fifteen medium resolution MGT5 STs matched those from human infections in Australia and globally. However, no STs matched between the chicken and human isolates at high resolution levels (MGT8 or MGT9), indicating the two STm populations were phylogenetically related but were unlikely to be directly epidemiologically linked. AMR genes were rare, with only a *bla*_TEM-1_ gene carried by a 95 kb IncI1 Alpha plasmid being identified in 20 isolates. In conclusion, subpopulations that were widespread in processing plants and had caused human infections were described using MGT5 STs. In this STM population, AMR was rare with only sporadic resistance to a single drug class observed. The genomic analysis of STm from chicken processing plants in this study provided insights into STm that contaminate meat chickens early in the food production chain.

## 1 Introduction

Non-Typhoidal *Salmonella* (NTS) is a common foodborne pathogen across the globe. *Salmonella enterica* serovar Typhimurium (STm) is the predominant NTS serovar causing foodborne infections in Australia. In recent decades, the escalation in reported instances of human salmonellosis cases and outbreaks has created a pressing public health concern (Ford et al., 2016; McLure et al., 2022). *Salmonella* primarily disseminates through contaminated food and contact with animal excrement or contaminated surfaces (Arnold et al., 2015; Moffatt et al., 2016). Infiltration of this pathogen into the food supply chain, especially during chicken processing, underscores the significance of investigating sources of contamination events (Fearnley et al., 2011; Ferrari et al., 2019; Stewart and Pavic, 2023). Strategies to prevent these events can then be developed to improve food safety and protect public health since the demand for chicken meat in Australia has increased significantly during the recent decades (Wong et al., 2015).

Integrated surveillance methods are important to understand and investigate outbreaks of *Salmonella* (Galanis et al., 2012; Sintchenko et al., 2012; Hu et al., 2021). Therefore, one of the most effective methodologies would be to detect the pathogen through the food production chain before causing infections and illness (Delgado-Suarez et al., 2018). Rapid, effective, and accurate identification of *Salmonella* populations is important to control outbreaks and disease transmission (Payne et al., 2020; Hu et al., 2021), and is vital for public health surveillance.

**Figure 1:**
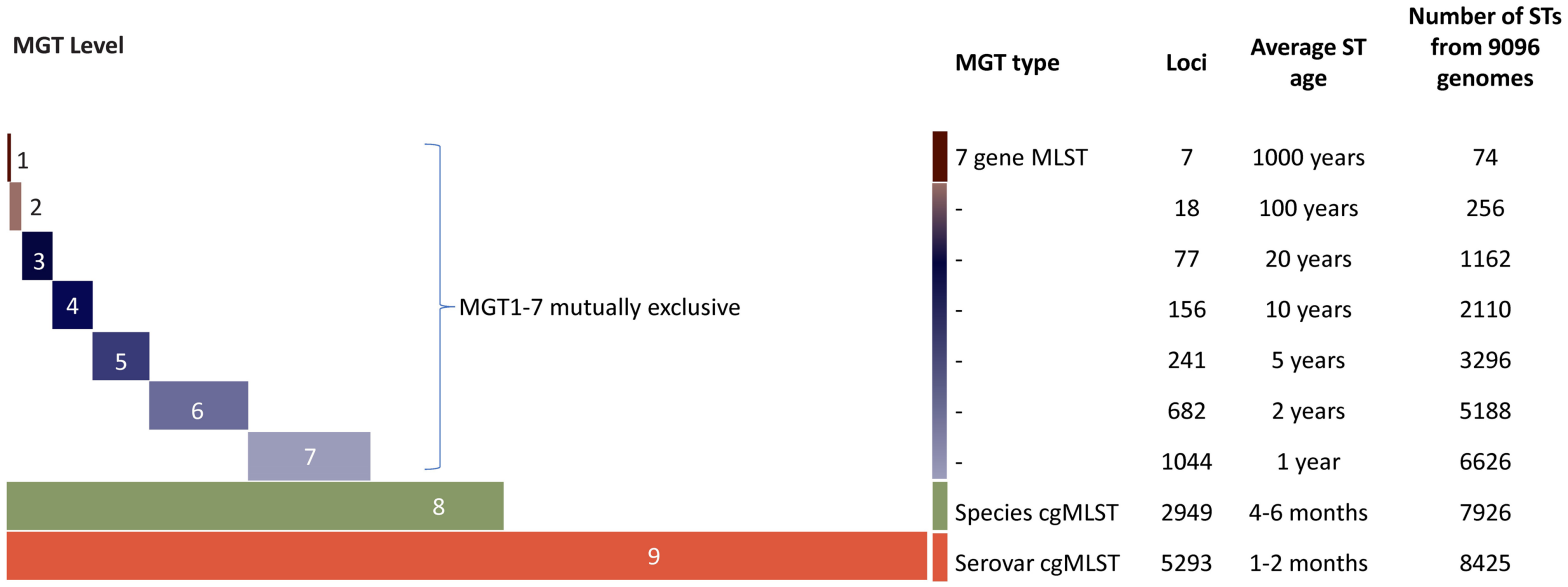
Illustrations of the multilevel genome typing (MGT) system from Payne et al. (2020). STm MGT scheme of 9 levels (represented by colored horizontal bars); the number of loci is increasing from MGT1 to MGT9 (represented by increasing length of the bars). The lowest resolution level, MGT1 is the classic seven gene MLST scheme and the highest resolution level, MGT9 made up of the cgMLST scheme of STm. MGT1–GT7 are mutually exclusive and composed of species cgMLST (MGT8). MGT8 (species cgMLST) is a subset of MGT9. The average ST age was defined as the average time for a new allele change to give rise to a new ST at a given MGT level. The increasing resolution was demonstrated using 9,096 genomes.

Multilevel genome typing (MGT) of STm is a publicly available whole genome sequencing (WGS) typing system (Payne et al., 2020; Kaur et al., 2022). It can be used to track STm spread and to understand evolutionary history of the pathogen (Payne et al., 2020). This typing method is based on multilocus sequence typing (MLST) (Maiden et al., 1998) and consisted of a set of nine MLST schemes progressively increasing in size and resolution (Figure 1) (Payne et al., 2020). Sequence types (STs) assigned at each MGT level can then be used to study the epidemiological distribution of STm populations (Payne et al., 2020). MGT1 is the traditional *S. enterica* MLST and has the lowest resolution whereas MGT8 is the core genome MLST (cgMLST) of the species, and MGT9 is the STm core genome MLST (serovar cgMLST) scheme (Payne et al., 2020). The highest resolution level, MGT9, is useful for outbreak investigation. By providing multiple levels of resolutions within a single typing system, MGT can accurately differentiate and type STm strains for both short and long-term epidemiology (Payne et al., 2020).

The emergence and dissemination of antimicrobial resistance (AMR) in NTS against several crucial antimicrobials like fluoroquinolones and extended spectrum cephalosporins are a significant threat to public health worldwide (Crump et al., 2011; WHO, 2014). The prevalence of ciprofloxacin resistance among *Salmonella* isolated from broilers was found to be high across 19 European Union member states (European Food Safety et al., 2021). In a recent international review, colistin resistance in *Salmonella* from poultry was found to be prevalent in both developing and developed countries, frequently surpassing 5% of isolated cases (Apostolakos and Piccirillo, 2018). Importantly, the rapid acquisition of AMR genes within plasmids potentially gives rise to multidrug-resistant bacteria that challenge treatment strategies (Ingle et al., 2021; Castaneda-Barba et al., 2023).

Within Australia, the restricted usage of antimicrobials in livestock and close monitoring of all fresh meat products (Barlow et al., 2015; Abraham et al., 2019) means that AMR incidence is minimal in *Salmonella* isolated from Australian farm animals (Barlow et al., 2015; Abraham et al., 2019, 2022). However, although there is a public health *Salmonella* surveillance program in Australia (OzFoodNetWorkingGroup, 2015), there is always a need to monitor AMR in farm animals for one health (Williamson et al., 2018; Abraham et al., 2019, 2022).

In this study, we were focused on early detection of STm along the chicken production chain. Therefore, the objective of this study was to assess the diversity and AMR carriage of *S.* Typhimurium in three chicken processing plants using whole genome sequencing and to investigate whether any particular strain types were associated with cases of human illness.

## 2 Materials and methods

### 2.1 Source of *Salmonella* Typhimurium isolates

In Australia there are two major meat chicken breeds, Cobb and Ross, and the Australian poultry industry harvest birds from 1,600 to 3,200 grams in dress weight. Microbiological screening of processed chicken carcasses is routinely performed at Birling Laboratory, this includes isolation of *Salmonella* using the ISO 6579 culture method with PCR confirmation for STm (Mooijman, 2018). The STm isolates were from three chicken processing plants in New South Wales (NSW) and were isolated from post chill carcasses as part of routine testing as required by the jurisdiction. A total of 379 STm isolates were collected as routine surveillance between January 2021 to December 2022 (Supplementary Table S2).

All samples tested were whole bird rinsates in accordance to AS 5013.20-2017. The standard Australian processing sampling plan is based upon number of samples processed with small plants (<100,000 samples) sampling 2 carcasses pre shift and large plants (>100,000 samples) sampling 1 in 25,000 carcasses. Whole carcasses were sent to service laboratories and the carcass rinse method was used to test for *Salmonella* presence/absence.

Carcasses were rinsed in 500 mL of buffered peptone water (BPW), the BPW was incubated overnight at 37°C. The non-selective BPW culture was then transferred into selective enrichment in Rappaport Vassiliades Broth (RVS) (Edwards Group),100 μL in 9.9 mL and Tetrathionate Hajna broth (Edwards Group), 1 mL in 9 mL broth. These broths were then incubated overnight at 42°C and 37°C, respectively. The selective broths were plated out on Hektoen and Xylose Lysine Deoxycholate (XLD) plates (Edwards Group), and incubated overnight at 37°C. Typical colonies were transferred onto ChromID *Salmonella* agar plates (Edwards Group), and incubated overnight at 37°C. Colonies with typical *Salmonella* features were then transferred to Nutrient Agar and incubated overnight at 37°C. The isolates were then confirmed as STm by serological grouping with O:5 and H:i antisera (Cell Biosciences). Further confirmation was carried out by a proprietary in-house amplicon based Next-generation sequencing (NGS) typing scheme. All 4,713 publicly available raw reads sets from Australian STm isolates were downloaded (5/4/2024) from Enterobase and NCBI (National Center for Biotechnology Information) and compared with *S.* Typhimurium isolated from chicken in this study.

### 2.2 DNA extraction, library preparation and sequencing

The genomic DNA was extracted using DNeasy Blood & Tissue Kits (QIAGEN) as per the manufacturer’s instructions. The sequencing libraries were prepared using Nextera XT DNA Library Prep Kit (Illumina) and sequenced on NextSeq 500 instrument using NextSeq 500/550 v2 mid output Kits (Illumina).

### 2.3 Multilevel genome typing

Raw reads of STm isolates were processed using MGT-Reads to Alleles as described in Payne et al. (2020). This script performs genome assembly, quality filtering, serotype confirmation (SISTR) and initial allele calling.^1^ Allele call files were then uploaded onto the MGT website to assign final allele numbers and MGT STs. Kraken2 (Wood et al., 2019) was used to identify any contamination with other species. The public STm genome data and MGT types were accessed through MGTdb (Kaur et al., 2022).^2^

### 2.4 Phylogenetic analysis

Phylogenetic tree of 379 STm isolates was built from allelic profiles of MGT9 STs using GrapeTree 1.5.0 (Zhou et al., 2018) using the RapidNJ algorithm (Simonsen et al., 2008). The visualization was executed utilizing the interactive mode provided by Grapetree.

Raw reads were used to build phylogenetic trees using core genome single nucleotide polymorphisms (SNPs). The reference genome sequence used was STm strain LT2 with complete genome (NC_003197.2) obtained from NCBI public databases in FASTA format. Snippy version 4.6.0^3^ was used to align the sequence reads to the reference genome and variants were identified. IQ-TREE^4^ multicore version 2.2.0.3 (COVID-edition for Linux 64-bit built Aug 2.2022) was used to construct the phylogenetic tree using the resulting core alignment generated from Snippy with 10,000 bootstrap pseudo replicates (Hoang et al., 2018). The results were visualized and annotated with metadata with iTol (version 6) interactive online tool (Letunic and Bork, 2021).

### 2.5 Antimicrobial resistance and plasmid specific genes

Antimicrobial resistance genes and mutations were detected from 379 assembled genomes using AbritAMR version 1.0.14^5^ using the AMRFinder Plus database (version 3.10.42) (Feldgarden et al., 2021; Sherry et al., 2023) and ResFinder (version 4.5.0) (Florensa et al., 2022).^6^ To identify plasmid specific AMR genes, assembly contigs were further annotated using Prokka (rapid prokaryotic genome annotation) version 1.14.6 (Seemann, 2014).^7^ The 379 STm isolates were screened for plasmids genes using plasmidfinder (Carattoli et al., 2014).

### 2.6 Nanopore sequencing

For isolates with AMR genes (20 isolates), nanopore sequencing was performed using a MinION [Oxford Nanopore Technologies, Oxford, UK (United Kingdom)]. Input genomic DNA was quantified using a Qubit fluorometer (Thermo Fisher Scientific, Waltham, MA, United States) and a NanoDrop spectrophotometer (Thermo Fisher Scientific). Then the long reads were assembled using microPIPE^8^ in combination with Illumina sequencing reads. Nucleotide blast (Camacho et al., 2009) analysis was performed using the NCBI nucleotide blast tool^9^ to identify similarities between the contigs with AMR gene and sequences available in the NCBI nucleotide database. Following the assembly of plasmid contigs, a phylogenetic analysis was conducted to elucidate the evolutionary relationship among the identified plasmids. The analysis was performed using Parsnp (Treangen et al., 2014)^10^ and the results were visualized using iTol (version 6) interactive online tool and Gingr (Treangen et al., 2014) Multiple plasmids were aligned using Clustal Omega (Sievers et al., 2011), and plasmid comparisons were visualized using BRIG (Alikhan et al., 2011). SNPs were identified by alignments within the plasmids. Repetitive regions were manually excluded. The isolates with AMR were screened for plasmids genes using plasmidfinder (Carattoli et al., 2014). Finally, BLAST was performed with identified plasmids gene sequences with the rest of the 373 genomic DNA sequences to detect the presence of plasmids.

## 3 Results

### 3.1 Multilevel genome typing and phylogenetic clustering of *Salmonella* Typhimurium chicken isolates

The 379 STm isolates from processed chicken carcasses were typed using the STm MGT scheme (Table 1). At MGT1 (seven-gene MLST) they were typed into two STs, with MGT1 ST19 and ST2066 representing 87.1% (330 isolates) and 12.9% (49 isolates) of the isolates, respectively. These isolates were further typed at higher MGT levels (Table 1). At MGT8 (species cgMLST), the isolates were divided into 204 ST with 1 to 23 isolates per ST. At MGT9 (serovar cgMLST), the isolates were divided into 257 STs with 1 to 16 isolates per ST.

**Table 1:** Multilevel genome typing of chicken *S.* Typhimurium isolates and comparison with human isolates.

MGT level	Total number of STs in chicken	Number of matching Human ST in Australia	Number of matching STs globally	Number of STs unique to chicken	% of isolates in matching STs	
					Chicken	Human
MGT1	2	1	2	0	87.1	97.1
MGT2	6	5	6	0	99.7	92.6
MGT3	18	11	10	7	84.7	81.6
MGT4	24	12	11	12	75.7	67.5
MGT5	41	15	6	26	68.3	52.6
MGT6	73	5	4	68	29.0	5.2
MGT7	106	2	1	104	7.9	0.1
MGT8	204	0	0	204	0.0	0.0
MGT9	257	0	0	257	0.0	0.0

Additionally, percentages of publicly available human isolates in MGTdb with the same STs found both globally and in Australia are also shown.

**Figure 2:**
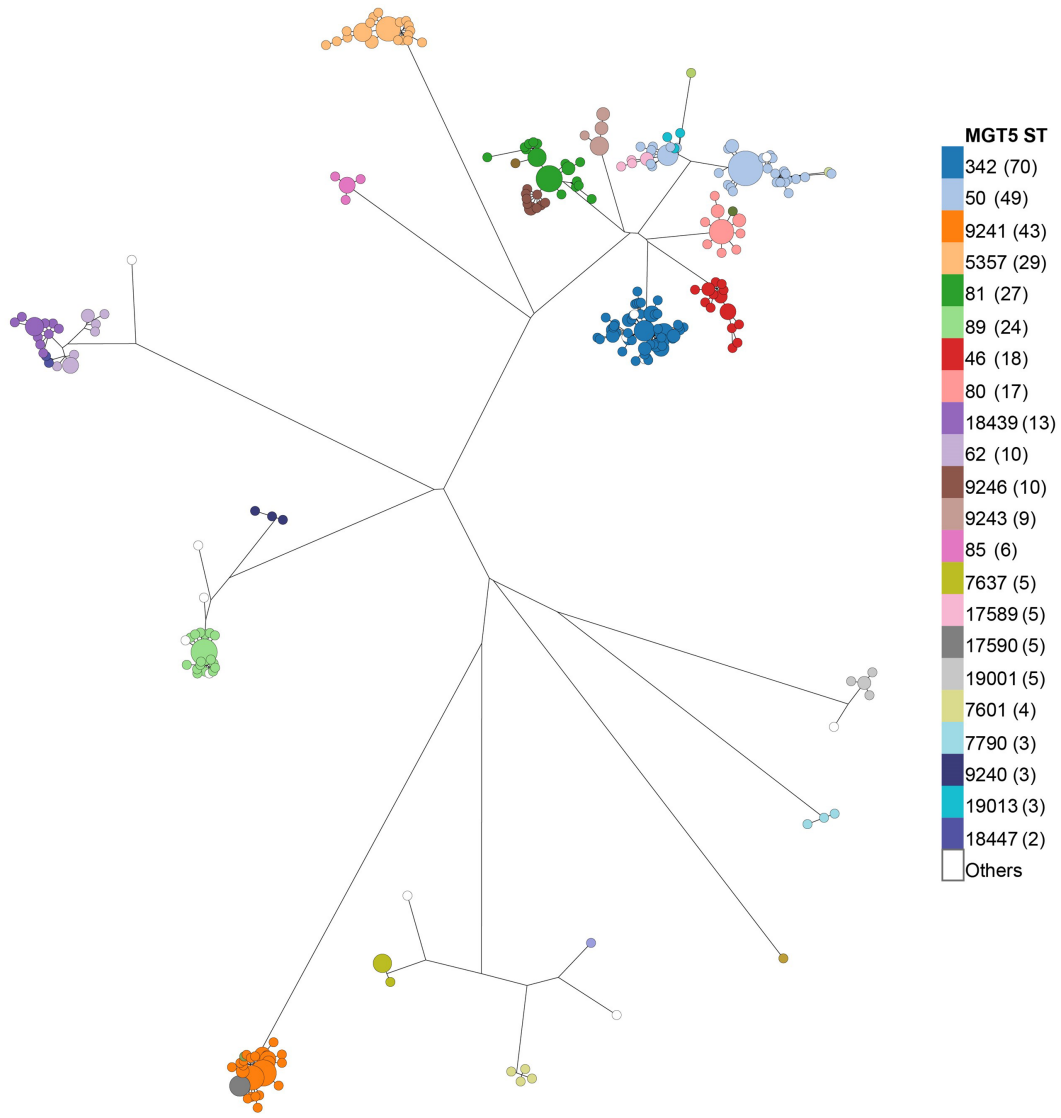
Population structure of chicken STm isolates at the MGT5 level. The phylogenetic tree was constructed using MGT9 allelic profiles. Each round dot at the tip of the tree represents an MGT9 ST. MGT5 STs were overlaid onto the tree per color legend to visualize MGT9 STs grouped by MGT5 STs and phylogenetic clustering of MGT5 STs. The Number in brackets after each ST in the color legend is the number of isolates of a given MGT5 ST.

Phylogenetic clustering of the STm isolates was performed using MGT9 allele profiles. STs at different MGT levels were mapped onto the tree (Figure 2; Supplementary Figures S3, S4) and MGT5 STs were found to best reflect the major phylogenetic clusters (Figure 2), compared to other MGT levels. Of the 41 MGT5 STs, 22 STs with more than one isolate contained 94.9% of the isolates, with MGT5 ST342 being the largest at 18.5% along with MGT5 ST50 at 12.9%, and MGT5 ST9241 at 11.3%. MGT8 and MGT9 STs were consistent with whole genome SNP analysis in phylogenetic grouping. At MGT8 and MGT9, 75.88 and 80.33% of STs were found to be in the same SNP type with identical resolution (Supplementary Table S3).

### 3.2 Epidemiological trends of the chicken STm isolates

**Figure 3:**
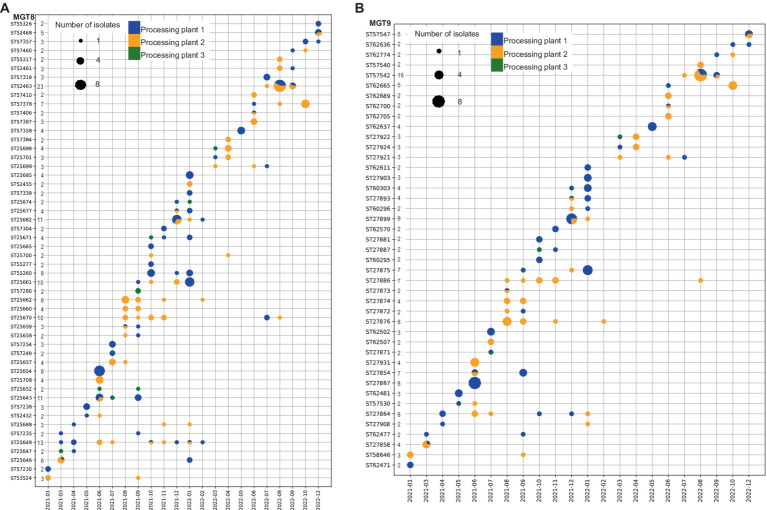
Temporal and spatial dynamics of isolates at MGT8 and MGT9 levels. Distribution of STs with multiple isolates per ST across the three processing plants and temporal patterns throughout the collection period. **(A)** At MGT8 level; **(B)** at MGT9 level. Y axis lists STs and the number of isolates while X axis marks the year and month of isolate collection. Note the discontinuity of months as months without isolates are not shown. The colors of the dots represent processing plants, and the size of the dots represents the number of isolates as shown in the legends.

The spatial and temporal trends of chicken STm isolates were examined using MGT5 (Supplementary Figure S2), MGT8 and MGT9 STs (Figure 3). The STm isolates were obtained from three different chicken processing plants which are from three different geographical locations with most of the isolates coming from plant 1 (52.2% of the isolates) (Supplementary Table S2). Of the 41 MGT5 STs, four with 42.2% of the total isolates (ST342, ST5357, ST46, and ST9241) were found in all three plants (Supplementary Figure S2A). The highest number of isolates and STs were between October 2021 to January 2022, which coincided with the hottest months of the year in Australia. There were persistent STs such as MGT5 ST342, MGT5 ST46, and MGT5 ST9241 which were found for more than 12 months while some MGT5 STs were only found in a single month (Supplementary Figure S2B).

At MGT8 (Figure 3A), 52 (59.9% of the isolates) of the 204 STs had more than 1 isolate and 1 ST (MGT9 ST25643) was shared across all the processing plants. Twenty-one MGT8 STs with 32.7% of the isolates were shared among two plants and 18 MGT8 STs with 29.5% of the isolates were shared between processing plant 1 and 2. Of these shared STs, MGT8 ST25670 and MGT8 ST25648 were sampled across 12 to 13 months from March 2021 to August 2022 while the rest were sampled across one to 11 months from March 2021 to December 2022. Plant specific STs were sampled across 1 to 4 months during the collection period.

At MGT9 (Figure 3B) only 43 (43.5% of the isolates) of the 257 STs had more than 1 isolate and similar to MGT8 STs, 21 MGT9 STs with 24.3% of the isolates were shared between two plants and none of the STs was shared among all the processing plants. At MGT9 20 STs represent a subset of their respective MGT8 STs demonstrating further resolution of MGT9 while the remaining STs contained the same isolates as their respective MGT8 STs. Of the 21 MGT9 STs found in multiple processing plants 18 STs, containing 22.4% of all isolates, were shared between processing plant 1 and 2. Of the shared STs, MGT9 ST27908, MGT9 ST27864, and MGT9 ST27866 were sampled across 10 to 13 months from April 2021 to August 2022 while the remaining 18 STs were sampled across 1 to 8 months throughout the collection period. All plant specific STs were sampled across 1 to 4 months.

### 3.3 Carriage of antibiotic resistance genes and plasmids

The 379 STm isolates were screened for AMR genes using AbritAMR and ResFinder. A *β*-lactamase gene, *bla*_TEM-1,_ was identified in 20 isolates that were collected in 2021 to 2022 (Table 2). They belonged to 15 MGT9 STs (Table 2). Of those MGT9 STs, ST27855, ST60296, and ST57540 had two isolates while MGT9 ST27854 had 3 isolates. To determine whether *bla*_TEM-1_ was carried by a plasmid or on the chromosome, we fully sequenced the genomes of six of the nine isolates from different MGT9 STs (Supplementary Table S2). The *bla*_TEM-1_ gene was found to be on an IncI1 Alpha plasmid in all six isolates (Table 2). All six isolates also carried an IncIFIB(S)/IncIFII(S) plasmid [similar to *S.* Typhimurium plasmid pSLT (AE006471)] and 3 isolates carried a Col156 plasmid [similar to *Salmonella enterica* Col156 plasmid (CP058808.1)]. BLAST analysis using complete sequences of all three plasmids extracted from the complete assembly of isolate 1014176-rl and confirmed that all the remaining 373 isolates with draft genomes in the study carried an IncIFIB(S)/IncIFII(S) plasmid while 40 isolates carried a Col156 plasmid and all 20 isolates with the *bla*_TEM-1_ gene carried the same IncI1 Alpha plasmid (Supplementary Table S2).

**Table 2:** Chicken *S.* Typhimurium isolates with *bla*_TEM-1_ gene and their characteristics.

Isolate	Collection year and month	Processing plant	Presence of AMR gene	MGT5 ST	MGT8 ST	MGT9 ST	IncI1 Alpha plasmid	Plasmid size (bp)
1233107-R1	2022-08	Plant 2	*bla*_TEM-1_	50	52463	62851	Not fully sequenced but detected	–
1236896-R1	2022-08	Plant 2	*bla*_TEM-1_	50	57378	62746	Not fully sequenced but detected	–
1258021-R1	2022-10	Plant 2	*bla*_TEM-1_	50	57378	62815	Not fully sequenced but detected	–
1136041-r1	2021-11	Plant 1	*bla*_TEM-1_	80	25713	27936	pSTm_1136041_93	93211
1146311-R1	2021-12	Plant 2	*bla*_TEM-1_	80	25661	60296	Not fully sequenced but detected	–
1153729-R1	2022-01	Plant 1	*bla*_TEM-1_	80	25661	60296	Not fully sequenced but detected	–
1151651-R1	2022-01	Plant 1	*bla*_TEM-1_	80	55297	60325	Not fully sequenced but detected	–
1014171-r1	2021-01	Plant 2	*bla*_TEM-1_	85	25644	27855	pSTm_1014171_94	94683
1014176-r1	2021-01	Plant 2	*bla*_TEM-1_	85	25706	27929	pSTm_1014176_93	93500
1014185-r1	2021-01	Plant 2	*bla*_TEM-1_	85	25645	27857	pSTm_1014185_94	94355
1014188-r1	2021-01	Plant 2	*bla*_TEM-1_	85	25644	27855	Not fully sequenced but detected	–
1107698-r1	2021-09	Plant 1	*bla*_TEM-1_	5357	25643	27854	Not fully sequenced but detected	–
1107699-r1	2021-09	Plant 1	*bla*_TEM-1_	5357	25643	27854	Not fully sequenced but detected	–
1107708-r1	2021-09	Plant 1	*bla*_TEM-1_	5357	25643	27854	pSTm_1107708_95	95679
1098187-r1	2021-08	Plant 2	*bla*_TEM-1_	7637	25660	27874	pSTm_1098187_94	94290
1232535-R1	2022-08	Plant 2	*bla*_TEM-1_	17589	52461	57540	Not fully sequenced but detected	–
1232536-R1	2022-08	Plant 2	*bla*_TEM-1_	17589	52461	57540	Not fully sequenced but detected	–
1249608-R1	2022-09	Plant 1	*bla*_TEM-1_	17589	52461	62773	Not fully sequenced but detected	–
1254317-R1	2022-10	Plant 2	*bla*_TEM-1_	17589	57528	62861	Not fully sequenced but detected	–
1254635-R1	2022-10	Plant 2	*bla*_TEM-1_	17589	57488	62809	Not fully sequenced but detected	–

**Figure 4:**
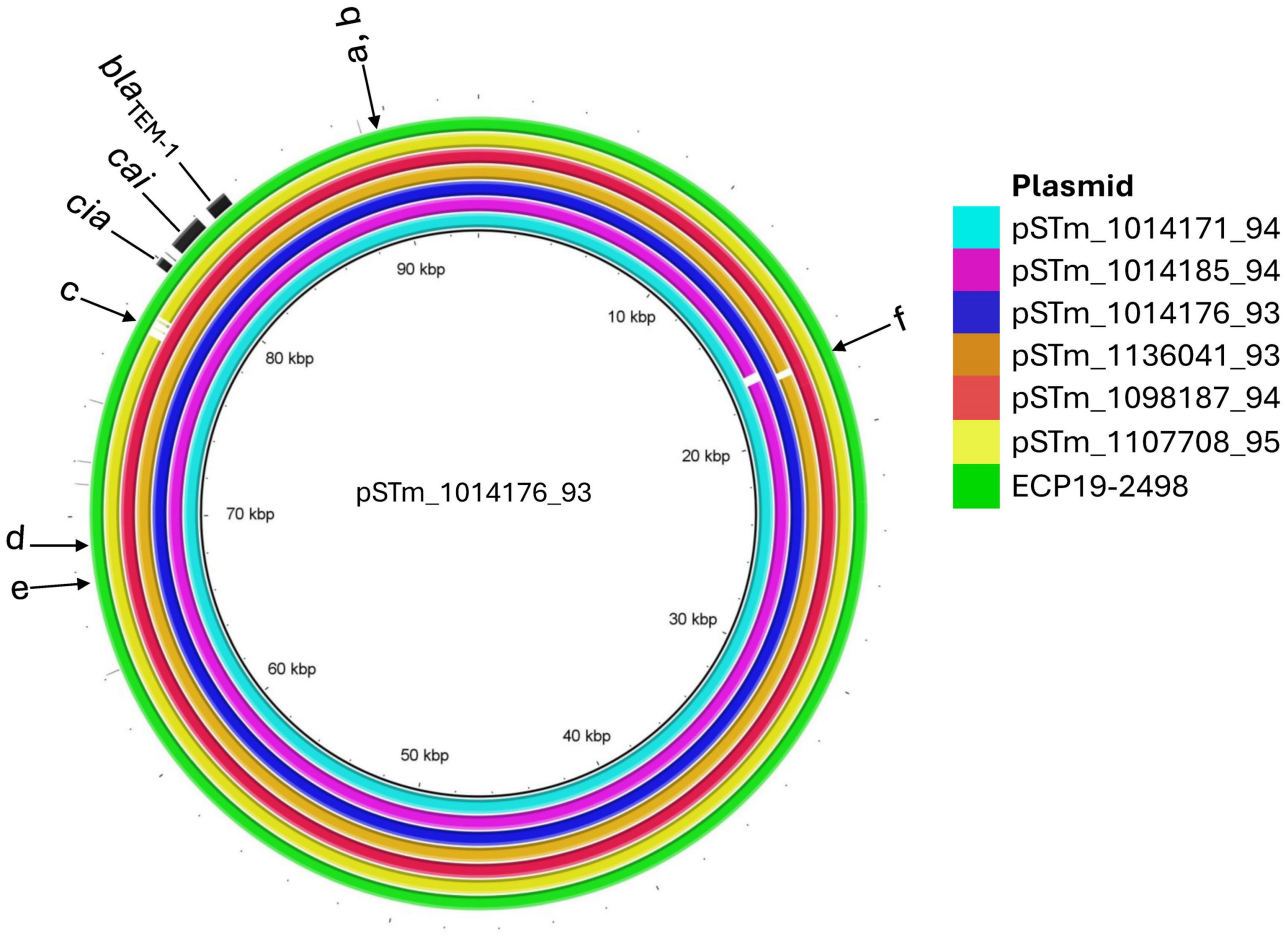
Multiple genome comparisons using BRIG with one of the IncI1 Alpha plasmids from this study, pSTm_1014176_93 as the reference. Colored rings indicate the sequence similarity between IncI1 Alpha plasmid genomes from the other isolates and *Escherichia coli* O157:H7 strain ECP19-2498 (CP066749.1) plasmid. In the figure, *bla*_TEM-1_, *cai* (colicin IA synthesis gene) and *cia* (colicin IA immunity protein synthesis gene) genes were annotated. Letters a-f indicates indels as follows: (a) IS629 (in pSTm_1014171_94), (b) IS1203 (in pSTm_1014185_94), (c) Antitoxin *ccdA* (in pSTm_1107708_95), (d) IS21 family transposase *istA*, (e) IS21-like element ISSso4 family helper ATPase *istB* (in ECP19-2498 plasmid) and (f) An indel region encoding Shufflon protein D was found in all the plasmid except for pSTm_1136041_93.

The IncI1 alpha plasmids from the six isolates were between 93,211 bp – 95,679 bp in size (Table 2) and were most similar (with more than 99% identity) to a plasmid from *Escherichia coli* ECP19-2498 (CP066749.1) which was 95,679 bp in size (Figure 4). Comparison of STm plasmids showed minor structural changes among them (Figure 4). pSTm_1014171_94 had an indel (89598–90332 bp) encoding an IS3 family insertion sequence (IS) IS629. pSTm_1014185_94 had an indel (89210–89845 bp) encoding an IS3 family IS IS1203. pSTm_1107708_95 had an indel (77797–78015 bp) encoding antitoxin *ccdA*. An indel encoding Shufflon protein D was found in all the plasmids except for pSTm_1136041_93. Colicin IA synthesis gene (*cai*) and colicin IA immunity protein synthesis gene (*cia*) were identified in six STm plasmids closely located with *bla*_TEM-1_ (less than 800 bp apart) (Figure 4). Within the *E coli* ECP19-2498 plasmid, there were two genes, *istA* (67923 bp–69458 bp) and *istB* (69475 bp–70230 bp) which were not found in the six STm plasmids. Apart from these indels, pairwise comparison of STm plasmids and ECP19-2498 plasmid differ by 1 to 6 SNPs.

### 3.4 Comparison of chicken STm isolates with historical Australian isolates in the MGT database

**Figure 5:**
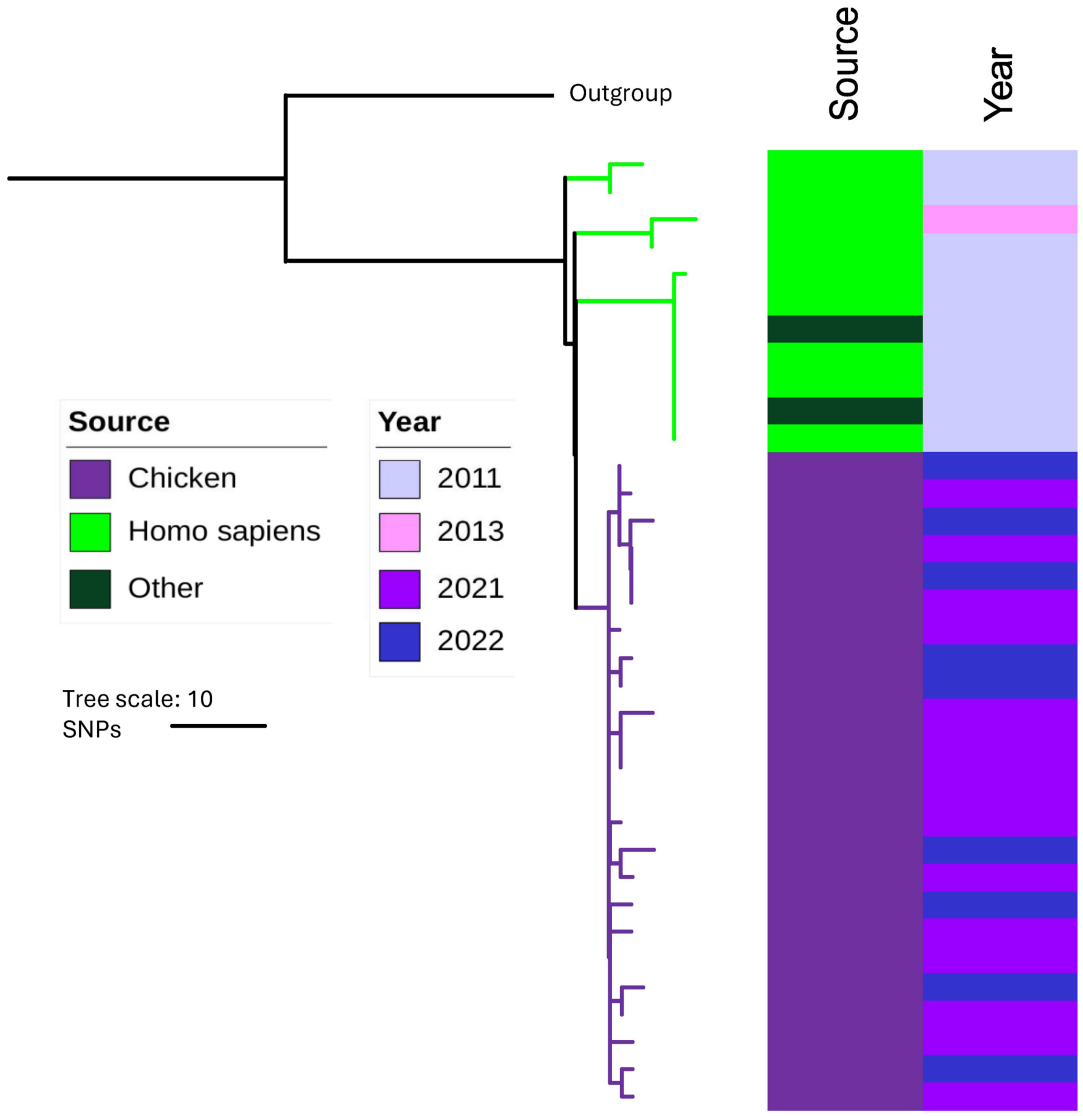
SNP based Phylogeny of isolates in MGT5 ST342. The colored strips on the right hand side represent source and the year of collection of the isolates. The outgroup used was an MGT5 ST5357 isolate. Chicken STm isolates (year 2021–2022) are represented by purple color and historical human isolates (year 2011–2013) in the MGT data base are represented by green color.

The chicken isolates were compared with historical Australian isolates in the MGT database at all MGT levels (Table 1). Among the 4,714 Australian isolates in the database, 3,728 were from humans, while the remaining isolates were from fecal matter (human or animal), animals, processed foods, raw food, and surfaces. These isolates had a collection year ranging from 1992 to 2020. No STs were identical at MGT8 and MGT9 levels between chicken isolates from this study and isolates in the database. At MGT5 (Supplementary Table S1), MGT6 and MGT7, there were 15, 5 and 2 STs that matched between our STm isolates and the database isolates (Table 1). Note that at MGT5, isolates of the same ST could have diverged by many years (Payne et al., 2020). As an example, we constructed an SNP based phylogeny of both human and chicken MGT5 ST342 isolates and showed that human and chicken derived isolates were in well separated branches of the tree (Figure 5). Thus, the human isolates shared a common ancestor with chicken isolates but with no direct epidemiological link.

We also compared STs of our chicken isolates at each MGT level with historical human STm isolates from other countries in the MGT database which had 16,374 human isolates (Table 1). No STs were identical at MGT6 to MGT9 between chicken STm from this study and isolates from other countries in the database (Supplementary Figure S1). At MGT5, two chicken STs, ST46 and ST3537, shared with human STs from other countries. MGT5 ST46 was found in New Zealand and United Kingdom while ST3537 was found in the United Kingdom (Supplementary Figure S1). All STs shared with other countries were also found in human isolates from Australia.

## 4 Discussion

Through contamination of meat chickens, *Salmonella* can pass down the food production chain to cause infections in humans (Ferrari et al., 2019; Van Immerseel et al., 2019). Meat chickens have a higher prevalence of *Salmonella* and one of the major sources of human exposure (McLure et al., 2022). However very little is known of STm from meat chickens in Australia as many outbreaks in Australia were caused by STm that can be traced back to contaminations of eggs or egg products (McWhorter et al., 2015; Moffatt et al., 2016; Ford et al., 2018; McLure et al., 2022). In this study, we sequenced 379 isolates obtained from 3 chicken processing plants to assess the genetic diversity of STm and their relationship to retrospective human isolates in Australia. Contemporary human isolate data from Australia were not publicly available, preventing direct comparison with meat chicken isolates of this study. Previous studies in NSW carcass processing facilities have shown low level of *Salmonella* cross-contamination between carcasses within the processing environment (Pavic et al., 2015). All processing facilities are required by the food regulators to frequently shut down operations to conduct cleaning which is verified by a complementary hygiene swabbing program using ATPase to assure no protein matter is present on food contact surfaces prior to start up (Pavic et al., 2015). Total viable counts are used to assure that the sanitizers and contact times are fit for purpose (Pavic et al., 2015). The cross contamination between carcasses during processing is possible (Pavic et al., 2015) but would be limited to birds slaughtered on the same day. The diversity of isolates seen in this study supports this finding as contamination from within the processing plant would likely be highly genetically homogenous. A more likely scenario is that STm sampled in this study is representative of a diverse set of endemic strains from local donor farms (<100 km from plants).

MGT for STm has 9 levels with scalable resolution (Payne et al., 2020). Lower MGT levels (MGT1 to MGT7) are useful for elucidating the longer-term epidemiological patterns, while the higher MGT levels (MGT8 and MGT9) are aimed for investigating short term epidemiological trends (Payne et al., 2020). Specifically, MGT8 and MGT9 corresponds to species-level cgMLST and serovar-level cgMLST, respectively (Payne et al., 2020). To showcase the application and difference in resolution of MGT8 and MGT9, we applied both to the STm isolates in this study. MGT9 offered the highest resolution and isolates of the same MGT9 ST were genetically nearly identical.

Using the increasing resolution of MGT levels the population of STm from processing plants can be compared with human isolates to identify the degree of relatedness between the two populations. By traditional MLST (MGT1), two STs, ST19 and ST2066 were observed. MGT1 ST19 is the predominant global ST while MGT1 ST2066 is relatively rare having been isolated in Australian human infections (Lan et al., 2009; Pang et al., 2012). By MGT5 there was considerable diversity with 41 STs, 15 of which overlapped with STs from human infections in Australia. Comparisons were also made with human STm from other countries, in particular, United Kingdom and United States as there were large numbers of STm sequenced. At MGT5, 2 STs were shared, these STs caused human infections in multiple countries including Australia. At MGT8 to MGT9, no STs were shared internationally and all the chicken STs were unique to Australia. Human isolates shared a common ancestor with chicken isolates in recent past, however there was no direct epidemiological link.

MGT typing revealed temporal and spatial trends of STm within and between processing plants. At MGT5, 4 STs were more prevalent and found across the collection period and were present in all three processing plants, suggesting that these STs were widely present across different farms while the STs that were specific to one plant were likely to be restricted to certain farms which supplied chickens to only one processing plant. At MGT9, there were few isolates with identical STs in a processing plant. Again, this diversity suggests that the STms were passed down from farms with the source live chicken rather than from persistent contamination of the processing plant. However, 43 of the 257 STs MGT9 STs (43.5% of the population) were sampled 2 to 16 times. About half of these STs were sampled from one processing plant. Five MGT9 STs were found in more than 1 plant from April 2021 to August 2022. This observation suggests a common vehicle of transmission that could include common breeder flocks, feed, equipment, and services across multiple farms. At MGT8 (species cgMLST), the pattern of distribution observed was similar to MGT9, but a higher percentage of the STs (59.9% of the population) contained more than 1 isolate. These findings highlight the usefulness of MGT8 and MGT9 STs as a high-resolution typing tool to potentially trace back the contamination of chicken products or infections in humans to its source at farm level.

The presence of AMR genes in the STm isolates of this study was very low, with 20 isolates carrying *bla*_TEM-1,_ a *β* lactamase gene conferring resistance to ampicillin. Rigorous regulation of antimicrobial usage in food animals within Australia has contributed to the low levels of AMR observed in bacteria originating from Australian livestock (Barlow et al., 2015; Abraham et al., 2019). The *bla*_TEM-1_ gene was found to be carried by an IncI1 Alpha plasmid in all *bla*_TEM-1_ positive isolates and the plasmid was highly similar to an *E. coli* plasmid. Gaining plasmids with resistance genes provides a competitive advantage in conditions with high antimicrobial usage (Alikhan et al., 2022). However, given the low antibiotic usage in Australia it is possible that the selection for this plasmid may be due to other plasmid encoded factors. The plasmid also carried colicin IA synthesis gene (*cai*) and colicin IA immunity protein synthesis gene (*cia*). It is possible that colicin production conferred a competitive advantage when competing with other microorganisms (Rendueles et al., 2014). IncI1 plasmids are frequently found in *Salmonella* from food animal sources as well as human infections (Smith et al., 2015; Kaldhone et al., 2019; Foley et al., 2021) and their capacity to disseminate AMR genes within enteric pathogens is well recognized (Smith et al., 2015; Kaldhone et al., 2019; Foley et al., 2021).

## 5 Conclusion

Genomic analysis of STm isolates from chicken processing plants in NSW provided insights into STm populations that contaminate chickens in the food production pipeline. This study also showcased the application of MGT in food production STm surveillance. There was a high diversity of STm with most isolates belonging to unique MGT9 STs. The diversity of STm from chicken processing plants was most likely a reflection of STm diversity at farm level. However, there were isolates from different sampling times or processing plants belonging to the same MGT9 STs, suggesting contamination of chickens by the same ST at its initial source or at the processing plants. Comparison of retrospective human isolates from Australia and other countries revealed that the chicken and human STm STs, in particular those from Australia, overlapped at MGT5. While the two STm populations were not identical, they were related and shared the most recent common ancestor. These findings will be useful for developing intervention strategies to reduce the transmission of STm down the food production chain to cause infections in humans.

## Data availability statement

The data presented in the study are deposited in the NCBI repository, https://www.ncbi.nlm.nih.gov/bioproject/PRJNA1119266.

## Author contributions

SB: Conceptualization, Data curation, Formal analysis, Investigation, Methodology, Project administration, Software, Visualization, Writing – original draft, Writing – review & editing. SW: Resources, Supervision, Validation, Writing – review & editing. JS: Resources, Writing – review & editing. MP: Conceptualization, Methodology, Project administration, Software, Supervision, Validation, Writing – review & editing. SK: Software, Validation, Writing – review & editing. QW: Resources, Writing – review & editing. VS: Resources, Writing – review & editing. AP: Resources, Supervision, Validation, Writing – review & editing. RL: Conceptualization, Funding acquisition, Methodology, Project administration, Supervision, Validation, Writing – review & editing.

## Footnotes

^1^ https://github.com/LanLab/MGT_reads2alleles

^2^ https://mgtdb.unsw.edu.au/typhimurium

^3^ https://github.Com/tseemann/snippy

^4^ https://github.com/iqtree/iqtree2

^5^ https://github.com/MDU-PHL/abritamr

^6^ https://bitbucket.org/genomicepidemiology/resfinder.git

^7^ https://github.com/tseemann/prokka

^8^ https://github.com/BeatsonLab-MicrobialGenomics/micropipe

^9^ https://blast.ncbi.nlm.nih.gov/Blast.cgi

^10^ https://github.com/marbl/parsnp
