# Supplementary material for: Genomic diversity of Salmonella enterica serovar Typhimurium isolated from chicken processing facilities in New South Wales, Australia
Source: Front Microbiol. 2024 Aug 14;15:1440777. doi: 10.3389/fmicb.2024.1440777 (PMC11349623; doi:10.3389/fmicb.2024.1440777)
Supplement: Supplementary file 1 [file Presentation_1.pdf]

# *Supplementary Material*

**Genomic diversity of *Salmonella enterica* serovar Typhimurium isolated from chicken processing facilities in New South Wales, Australia**

**Samitha Bandaranayake[1], Sarah Williamson[2], Jack Stewart[1,2], Michael Payne[1], Sandeep Kaur[1], Qinning Wang[3], Vitali Sintchenko[3,4], Anthony Pavic[2], Ruiting Lan[1*]**

1. School of Biotechnology and Biomolecular Sciences, University of New South Wales, New South Wales 2052, Australia.
2. Birling Laboratories, Bringelly, New South Wales 2556, Australia.
3. Centre for Infectious Diseases and Microbiology-Laboratory Services, Institute of Clinical Pathology and Medical Research, NSW Health Pathology, Westmead Hospital, New South Wales 2145, Australia
4. Marie Bashir Institute for Infectious Diseases and Biosecurity, Sydney Medical School, University of Sydney, New South Wales 2006, Australia.

* Corresponding author: r.lan@unsw.edu.au

## Supplementary tables and figures

Supplementary table S1: Comparison of Chicken MGT5 STs with historical Australian human MGT5 STs in MGT database

| MGT5 ST | Number of chicken isolates | Number of Australian human isolates |
|---|---|---|
| 46 | 18 | 157 |
| 49 | 1 | 1 |
| 50 | 49 | 25 |
| 62 | 10 | 543 |
| 80 | 17 | 11 |
| 81 | 27 | 763 |
| 85 | 6 | 194 |
| 89 | 24 | 39 |
| 342 | 70 | 9 |
| 3537 | 1 | 133 |
| 5357 | 29 | 8 |
| 7408 | 1 | 1 |
| 7595 | 1 | 32 |
| 7601 | 4 | 44 |
| 9249 | 1 | 1 |

Supplementary table S2: Summary of results from Chicken STm isolates used in the study.

| Isolate | Collection month and the year | Processing plant | IncIFIB(S)/IncIFII(S) plasmid | Col156 plasmid | IncI1 plasmid | MGT5 ST | MGT8 ST | MGT9 ST | Accession numbers: illumina reads | Accession numbers: nanopore reads |
|---|---|---|---|---|---|---|---|---|---|---|
| 1108498-r1 | Sep-21 | Processing plant 1 | present | | | 46 | 25659 | 27877 | SRR29260552 | |
| 1091620-r1 | Aug-21 | Processing plant 1 | present | | | 46 | 25659 | 27873 | SRR29260551 | |
| 1120145-r1 | Oct-21 | Processing plant 1 | present | | | 46 | 25664 | 27880 | SRR29260504 | |
| 1118132-r1 | Oct-21 | Processing plant 1 | present | | | 46 | 25665 | 27881 | SRR29260589 | |
| 1118508-r1 | Oct-21 | Processing plant 1 | present | | | 46 | 25665 | 27881 | SRR29260258 | |
| 1179380-r1 | Mar-22 | Processing plant 2 | present | | | 46 | 25698 | 27921 | SRR29260247 | |
| 1118145-r1 | Oct-21 | Processing plant 2 | present | | | 46 | 25668 | 27884 | SRR29260236 | |
| 1144082-r1 | Dec-21 | Processing plant 1 | present | | | 46 | 25674 | 27890 | SRR29260583 | |
| 1160984-r1 | Jan-22 | Processing plant 3 | present | | | 46 | 25674 | 27902 | SRR29260572 | |
| 1125240-r1 | Oct-21 | Processing plant 1 | present | | | 46 | 25711 | 27934 | SRR29260561 | |
| 1032912-r1 | Mar-21 | Processing plant 2 | present | present | | 342 | 25646 | 27858 | SRR29260550 | |
| 1119002-r1 | Oct-21 | Processing plant 1 | present | | | 342 | 25648 | 27864 | SRR29260539 | |
| 1148669-r1 | Dec-21 | Processing plant 1 | present | | | 342 | 25648 | 27864 | SRR29260528 | |
| 1074243-r1 | Jun-21 | Processing plant 2 | present | | | 342 | 25648 | 27864 | SRR29260517 | |
| 1043610-r1 | Apr-21 | Processing plant 1 | present | | | 342 | 25648 | 27864 | SRR29260410 | |
| 1161019-r1 | Jan-22 | Processing plant 1 | present | | | 342 | 25646 | 27938 | SRR29260399 | |
| 1162336-r1 | Jan-22 | Processing plant 2 | present | present | | 342 | 25648 | 27864 | SRR29260322 | |
| 1163353-r1 | Feb-22 | Processing plant 1 | present | | | 342 | 25648 | 27910 | SRR29260311 | |
| 1164638-r1 | Feb-22 | Processing plant 1 | present | | | 342 | 25689 | 27909 | SRR29260300 | |
| 1042743-r1 | Mar-21 | Processing plant 1 | present | present | | 342 | 25648 | 27860 | SRR29260515 | |
| 1043604-r1 | Apr-21 | Processing plant 1 | present | | | 342 | 25649 | 27861 | SRR29260503 | |
| 1085945-r1 | Jul-21 | Processing plant 2 | present | | | 342 | 25657 | 27870 | SRR29260492 | |

| | | | | | | | | | | |
|---|---|---|---|---|---|---|---|---|---|---|
| 1120752-r1 | Oct-21 | Processing plant 2 | present | | | 342 | 25667 | 27883 | SRR29260287 | |
| 1127994-r1 | Nov-21 | Processing plant 2 | present | present | | 342 | 25669 | 27885 | SRR29260276 | |
| 1159000-r1 | Jan-22 | Processing plant 1 | present | | | 342 | 25671 | 27907 | SRR29260265 | |
| 1153096-r1 | Jan-22 | Processing plant 1 | present | | | 342 | 25671 | 27894 | SRR29260480 | |
| 1126391-r1 | Oct-21 | Processing plant 3 | present | | | 342 | 25671 | 27887 | SRR29260469 | |
| 1134930-r1 | Nov-21 | Processing plant 1 | present | | | 342 | 25672 | 27888 | SRR29260458 | |
| 1149183-r1 | Dec-21 | Processing plant 1 | present | | | 9244 | 25683 | 27900 | SRR29260221 | |
| 1157031-r1 | Jan-22 | Processing plant 2 | present | | | 342 | 25688 | 27908 | SRR29260600 | |
| 1162708-r1 | Jan-22 | Processing plant 1 | present | | | 342 | 25648 | 27916 | SRR29260588 | |
| 1192009-r1 | Apr-22 | Processing plant 2 | present | | | 342 | 25700 | 27923 | SRR29260381 | |
| 1113827-r1 | Sep-21 | Processing plant 1 | present | | | 9248 | 25710 | 27933 | SRR29260370 | |
| 1042584-r1 | Mar-21 | Processing plant 3 | present | | | 342 | 25647 | 27859 | SRR29260359 | |
| 1043606-r1 | Apr-21 | Processing plant 1 | present | | | 342 | 25650 | 27862 | SRR29260444 | |
| 1133494-r1 | Nov-21 | Processing plant 1 | present | | | 342 | 25671 | 27887 | SRR29260433 | |
| 1041522-r1 | Mar-21 | Processing plant 1 | present | | | 9247 | 25707 | 27930 | SRR29260422 | |
| 1157332-r1 | Jan-22 | Processing plant 1 | present | | | 342 | 25686 | 27905 | SRR29260347 | |
| 1067914-r1 | Jun-21 | Processing plant 1 | present | | | 81 | 25654 | 27867 | SRR29260336 | |
| 1064642-r1 | May-21 | Processing plant 1 | present | | | 81 | 25655 | 27868 | SRR29260325 | |
| 1175623-r1 | Mar-22 | Processing plant 1 | present | | | 9246 | 25696 | 27919 | SRR29260257 | |
| 1180374-r1 | Mar-22 | Processing plant 1 | present | | | 81 | 25702 | 27925 | SRR29260256 | |
| 1193754-r1 | Apr-22 | Processing plant 1 | present | | | 9246 | 25703 | 27926 | SRR29260255 | |
| 1192221-r1 | Apr-22 | Processing plant 1 | present | | | 81 | 25704 | 27927 | SRR29260254 | |
| 1014171-r1 | Jan-21 | Processing plant 2 | present | present | present | 85 | 25644 | 27855 | SRR29260253 | SAMN43006511 |
| 1014176-r1 | Jan-21 | Processing plant 2 | present | present | present | 85 | 25706 | 27929 | SRR29260252 | SAMN43006506 |
| 1014185-r1 | Jan-21 | Processing plant 2 | present | present | present | 85 | 25645 | 27857 | SRR29260251 | SAMN43006507 |
| 1107954-r1 | Sep-21 | Processing plant 2 | present | present | | 85 | 25644 | 27855 | SRR29260250 | |
| 1014188-r1 | Jan-21 | Processing plant 2 | present | present | | 85 | 25644 | 27855 | SRR29260248 | |
| 1108497-r1 | Sep-21 | Processing plant 1 | present | | | 80 | 25661 | 27875 | SRR29260246 | |
| 1156550-r1 | Jan-22 | Processing plant 1 | present | | | 80 | 25661 | 27875 | SRR29260245 | |
| 1147700-r1 | Dec-21 | Processing plant 1 | present | | | 80 | 25678 | 27895 | SRR29260244 | |
| 1148781-r1 | Dec-21 | Processing plant 2 | present | | | 80 | 25661 | 27875 | SRR29260243 | |
| 1110926-r1 | Sep-21 | Processing plant 1 | present | | | 80 | 25663 | 27879 | SRR29260242 | |
| 1136041-r1 | Nov-21 | Processing plant 1 | present | | present | 80 | 25713 | 27936 | SRR29260241 | SAMN43006510 |
| 1096138-r1 | Aug-21 | Processing plant 2 | present | | | 80 | 25658 | 27872 | SRR29260240 | |
| 1144081-r1 | Dec-21 | Processing plant 1 | present | | | 89 | 25675 | 27891 | SRR29260239 | |
| 1149182-r1 | Dec-21 | Processing plant 1 | present | | | 89 | 25682 | 27899 | SRR29260238 | |
| 1148709-r1 | Dec-21 | Processing plant 1 | present | | | 89 | 25682 | 27899 | SRR29260237 | |
| 1146627-r1 | Dec-21 | Processing plant 1 | present | | | 89 | 25682 | 27899 | SRR29260235 | |
| 1149577-r1 | Dec-21 | Processing plant 1 | present | | | 89 | 25682 | 27899 | SRR29260234 | |
| 1172206-r1 | Feb-22 | Processing plant 1 | present | | | 89 | 25682 | 27912 | SRR29260233 | |
| 1175626-r1 | Mar-22 | Processing plant 1 | present | | | 89 | 25697 | 27920 | SRR29260232 | |
| 1172989-r1 | Feb-22 | Processing plant 1 | present | | | 89 | 25695 | 27918 | SRR29260231 | |
| 1158726-r1 | Jan-22 | Processing plant 2 | present | | | 89 | 25693 | 27915 | SRR29260230 | |
| 1146625-r1 | Dec-21 | Processing plant 1 | present | | | 89 | 25679 | 27896 | SRR29260229 | |
| 1149180-r1 | Dec-21 | Processing plant 1 | present | | | 89 | 25681 | 27898 | SRR29260228 | |
| 1172207-r1 | Feb-22 | Processing plant 1 | present | | | 89 | 25691 | 27913 | SRR29260227 | |
| 1142710-r1 | Dec-21 | Processing plant 2 | present | | | 9242 | 25676 | 27892 | SRR29260584 | |
| 1161017-r1 | Jan-22 | Processing plant 1 | present | | | 89 | 25684 | 27901 | SRR29260582 | |
| 1157329-r1 | Jan-22 | Processing plant 1 | present | | | 89 | 25687 | 27906 | SRR29260581 | |
| 1171893-r1 | Feb-22 | Processing plant 2 | present | | | 9245 | 25692 | 27914 | SRR29260580 | |
| 1042579-r1 | Mar-21 | Processing plant 3 | present | | | 7601 | 25639 | 27850 | SRR29260577 | |
| 1080121-r1 | Jul-21 | Processing plant 1 | present | | | 5357 | 25643 | 27871 | SRR29260569 | |
| 1072974-r1 | Jun-21 | Processing plant 1 | present | | | 5357 | 25653 | 27866 | SRR29260568 | |
| 1107698-r1 | Sep-21 | Processing plant 1 | present | | present | 5357 | 25643 | 27854 | SRR29260567 | |
| 1107699-r1 | Sep-21 | Processing plant 1 | present | | present | 5357 | 25643 | 27854 | SRR29260563 | |
| 1107708-r1 | Sep-21 | Processing plant 1 | present | | present | 5357 | 25643 | 27854 | SRR29260562 | SAMN43006509 |
| 1056847-r1 | May-21 | Processing plant 2 | present | | | 5357 | 25651 | 27863 | SRR29260560 | |
| 1140458-r1 | Nov-21 | Processing plant 1 | present | | | 5357 | 25673 | 27889 | SRR29260559 | |
| 1069171-r1 | Jun-21 | Processing plant 2 | present | | | 5357 | 25708 | 27931 | SRR29260558 | |

| | | | | | | | | | | |
|---|---|---|---|---|---|---|---|---|---|---|
| 1129727-r1 | Nov-21 | Processing plant 3 | present | | | 5357 | 25712 | 27935 | SRR29260557 | |
| 1084875-r1 | Jul-21 | Processing plant 1 | present | | | 5357 | 25709 | 27932 | SRR29260556 | |
| 1108965-r1 | Sep-21 | Processing plant 3 | present | | | 9240 | 25652 | 27878 | SRR29260555 | |
| 1075522-r1 | Jun-21 | Processing plant 3 | present | | | 9240 | 25652 | 27865 | SRR29260554 | |
| 1081240-r1 | Jul-21 | Processing plant 3 | present | | | 7790 | 25656 | 27869 | SRR29260553 | |
| 1098186-r1 | Aug-21 | Processing plant 2 | present | | | 7637 | 25660 | 27874 | SRR29260324 | |
| 1098187-r1 | Aug-21 | Processing plant 2 | present | | present | 7637 | 25660 | 27874 | SRR29260323 | SAMN43006508 |
| 1121167-r1 | Oct-21 | Processing plant 1 | present | | | 7637 | 25666 | 27882 | SRR29260549 | |
| 1131325-r1 | Nov-21 | Processing plant 2 | present | | | 9241 | 25670 | 27886 | SRR29260548 | |
| 1100428-r1 | Aug-21 | Processing plant 2 | present | | | 9241 | 25662 | 27876 | SRR29260547 | |
| 1127499-r1 | Nov-21 | Processing plant 2 | present | | | 9241 | 25662 | 27876 | SRR29260546 | |
| 1187841-r1 | Apr-22 | Processing plant 2 | present | | | 9241 | 25701 | 27924 | SRR29260545 | |
| 1113342-r1 | Sep-21 | Processing plant 2 | present | | | 9241 | 25662 | 27876 | SRR29260544 | |
| 1170821-r1 | Feb-22 | Processing plant 2 | present | | | 9241 | 25662 | 27876 | SRR29260543 | |
| 1185697-r1 | Mar-22 | Processing plant 1 | present | | | 9241 | 25701 | 27924 | SRR29260542 | |
| 1185689-r1 | Mar-22 | Processing plant 3 | present | | | 9241 | 25699 | 27922 | SRR29260541 | |
| 1190384-r1 | Apr-22 | Processing plant 2 | present | | | 9241 | 25705 | 27928 | SRR29260540 | |
| 1160606-r1 | Jan-22 | Processing plant 1 | present | | | 9243 | 25685 | 27903 | SRR29260538 | |
| 1147147-r1 | Dec-21 | Processing plant 2 | present | | | 9243 | 25677 | 27893 | SRR29260537 | |
| 1150358-r1 | Dec-21 | Processing plant 1 | present | | | 9243 | 25677 | 27893 | SRR29260536 | |
| 1160607-r1 | Jan-22 | Processing plant 1 | present | | | 9243 | 25685 | 27903 | SRR29260535 | |
| 1160605-r1 | Jan-22 | Processing plant 1 | present | | | 9243 | 25685 | 27904 | SRR29260534 | |
| 1160608-r1 | Jan-22 | Processing plant 1 | present | | | 9243 | 25685 | 27903 | SRR29260533 | |
| 1152325-r1 | Jan-22 | Processing plant 1 | present | present | | 9243 | 25677 | 27893 | SRR29260532 | |
| 1166720-r1 | Feb-22 | Processing plant 1 | present | | | 9243 | 25694 | 27917 | SRR29260531 | |
| 1146091-r1 | Dec-21 | Processing plant 1 | present | | | 3537 | 25680 | 27897 | SRR29260530 | |
| 1162706-r1 | Jan-22 | Processing plant 1 | present | | | 7408 | 25690 | 27911 | SRR29260529 | |
| 1143254-r1 | Dec-21 | Processing plant 1 | present | | | 9249 | 25714 | 27937 | SRR29260527 | |
| 1157582-R1 | Jan-22 | Processing plant 2 | present | | | 46 | 57347 | 62624 | SRR29260526 | |
| 1218948-R1 | Jul-22 | Processing plant 1 | present | | present | 46 | 57421 | 62719 | SRR29260525 | |
| 1279846-R1 | Dec-22 | Processing plant 1 | present | | | 46 | 55326 | 62826 | SRR29260524 | |
| 1090736-R1 | Aug-21 | Processing plant 2 | present | | | 46 | 25659 | 27873 | SRR29260523 | |
| 1220975-R1 | Jul-22 | Processing plant 1 | present | | present | 46 | 25698 | 27921 | SRR29260522 | |
| 1215918-R1 | Jun-22 | Processing plant 2 | present | | present | 46 | 25698 | 27921 | SRR29260521 | |
| 1212467-R1 | Jun-22 | Processing plant 1 | present | | | 50 | 57378 | 62665 | SRR29260520 | |
| 1257309-R1 | Oct-22 | Processing plant 2 | present | | | 50 | 57378 | 62665 | SRR29260519 | |
| 1254582-R1 | Oct-22 | Processing plant 2 | present | | | 50 | 57378 | 62665 | SRR29260518 | |
| 1255255-R1 | Oct-22 | Processing plant 2 | present | | | 50 | 57378 | 62665 | SRR29260420 | |
| 1257314-R1 | Oct-22 | Processing plant 2 | present | | | 50 | 57378 | 62665 | SRR29260419 | |
| 1234846-R1 | Aug-22 | Processing plant 2 | present | | | 50 | 55317 | 62742 | SRR29260418 | |
| 1236897-R1 | Aug-22 | Processing plant 2 | present | | | 50 | 57439 | 62747 | SRR29260417 | |
| 1249608-R1 | Sep-22 | Processing plant 1 | present | | present | 17589 | 52461 | 62773 | SRR29260416 | |
| 1254617-R1 | Oct-22 | Processing plant 2 | present | | | 50 | 57461 | 62775 | SRR29260415 | |
| 1257323-R1 | Oct-22 | Processing plant 1 | present | | | 19013 | 57491 | 62812 | SRR29260414 | |
| 1258021-R1 | Oct-22 | Processing plant 2 | present | | present | 50 | 57378 | 62815 | SRR29260413 | |
| 1260090-R1 | Oct-22 | Processing plant 2 | present | | | 50 | 57493 | 62816 | SRR29260412 | |
| 1260093-R1 | Oct-22 | Processing plant 2 | present | | | 50 | 57494 | 62817 | SRR29260411 | |
| 1254317-R1 | Oct-22 | Processing plant 2 | present | | present | 17589 | 57528 | 62861 | SRR29260409 | |
| 1233549-R1 | Aug-22 | Processing plant 2 | present | | | 50 | 55317 | 60348 | SRR29260408 | |
| 1236896-R1 | Aug-22 | Processing plant 2 | present | | present | 50 | 57378 | 62746 | SRR29260407 | |
| 1254635-R1 | Oct-22 | Processing plant 2 | present | | present | 17589 | 57488 | 62809 | SRR29260406 | |
| 1257315-R1 | Oct-22 | Processing plant 1 | present | | | 19013 | 57490 | 62811 | SRR29260405 | |
| 1257325-R1 | Oct-22 | Processing plant 1 | present | | | 19013 | 57492 | 62813 | SRR29260404 | |
| 1055096-R1 | May-21 | Processing plant 1 | present | present | | 62 | 57238 | 62481 | SRR29260403 | |
| 1055095-R1 | May-21 | Processing plant 1 | present | present | | 62 | 57238 | 62481 | SRR29260402 | |
| 1055094-R1 | May-21 | Processing plant 1 | present | present | | 62 | 57238 | 62481 | SRR29260401 | |
| 1100339-R1 | Aug-21 | Processing plant 1 | present | present | | 62 | 57271 | 62522 | SRR29260400 | |
| 1035220-R1 | Mar-21 | Processing plant 2 | present | | | 342 | 25646 | 27858 | SRR29260398 | |
| 1041161-R1 | Mar-21 | Processing plant 1 | present | | present | 342 | 25646 | 27858 | SRR29260397 | |

| | | | | | | | | | |
|---|---|---|---|---|---|---|---|---|---|
| 1043992-R1 | Apr-21 | Processing plant 1 | present | | present | 342 | 25648 | 27864 | SRR29260396 |
| 1034783-R1 | Mar-21 | Processing plant 2 | present | | | 342 | 25646 | 27858 | SRR29260395 |
| 1074243-R2 | Jun-21 | Processing plant 2 | present | | | 342 | 25648 | 27864 | SRR29260394 |
| 1076660-R1 | Jul-21 | Processing plant 2 | present | | | 342 | 25648 | 27864 | SRR29260393 |
| 1046147-R1 | Apr-21 | Processing plant 1 | present | | | 342 | 25688 | 27908 | SRR29260392 |
| 1039790-R1 | Mar-21 | Processing plant 1 | present | | | 342 | 57234 | 62476 | SRR29260391 |
| 1039795-R1 | Mar-21 | Processing plant 1 | present | | | 342 | 57235 | 62477 | SRR29260390 |
| 1109460-R1 | Sep-21 | Processing plant 1 | present | | | 342 | 57235 | 62477 | SRR29260389 |
| 1041525-R1 | Apr-21 | Processing plant 1 | present | | | 342 | 25647 | 62479 | SRR29260321 |
| 1083028-R1 | Jul-21 | Processing plant 1 | present | | | 342 | 57254 | 62502 | SRR29260320 |
| 1083029-R1 | Jul-21 | Processing plant 1 | present | | | 342 | 57254 | 62502 | SRR29260319 |
| 1092399-R1 | Aug-21 | Processing plant 2 | present | | | 342 | 25657 | 62509 | SRR29260318 |
| 1108466-R1 | Sep-21 | Processing plant 2 | present | | | 342 | 57283 | 62537 | SRR29260317 |
| 1119848-R1 | Oct-21 | Processing plant 2 | present | | present | 342 | 25700 | 62546 | SRR29260316 |
| 1126362-R1 | Oct-21 | Processing plant 2 | present | | | 342 | 57293 | 62556 | SRR29260315 |
| 1126367-R1 | Oct-21 | Processing plant 2 | present | | | 342 | 57294 | 62557 | SRR29260314 |
| 1126946-R1 | Nov-21 | Processing plant 2 | present | | | 342 | 57295 | 62558 | SRR29260313 |
| 1133624-R1 | Nov-21 | Processing plant 2 | present | | | 342 | 25688 | 62561 | SRR29260312 |
| 1135049-R1 | Nov-21 | Processing plant 2 | present | | | 342 | 25648 | 62567 | SRR29260310 |
| 1135674-R1 | Nov-21 | Processing plant 2 | present | | | 342 | 57302 | 62568 | SRR29260309 |
| 1137297-R1 | Nov-21 | Processing plant 1 | present | | | 342 | 57304 | 62570 | SRR29260308 |
| 1131352-R1 | Nov-21 | Processing plant 1 | present | | | 342 | 57304 | 62570 | SRR29260307 |
| 1039852-R1 | Mar-21 | Processing plant 2 | present | | | 342 | 57463 | 62777 | SRR29260306 |
| 1154135-R1 | Jan-22 | Processing plant 1 | present | | | 342 | 57339 | 62611 | SRR29260305 |
| 1154114-R1 | Jan-22 | Processing plant 1 | present | | | 342 | 57339 | 62611 | SRR29260304 |
| 1155922-R1 | Jan-22 | Processing plant 2 | present | | | 342 | 52455 | 62619 | SRR29260303 |
| 1040626-R1 | Mar-21 | Processing plant 1 | present | | | 342 | 57236 | 62478 | SRR29260302 |
| 1045460-R1 | Apr-21 | Processing plant 1 | present | | | 342 | 57237 | 62480 | SRR29260301 |
| 1085043-R1 | Jul-21 | Processing plant 2 | present | | | 342 | 57257 | 62505 | SRR29260299 |
| 1085049-R1 | Jul-21 | Processing plant 2 | present | | | 342 | 25657 | 62507 | SRR29260298 |
| 1085493-R1 | Jul-21 | Processing plant 2 | present | | | 342 | 25657 | 62507 | SRR29260297 |
| 1085045-R1 | Jul-21 | Processing plant 2 | present | | | 342 | 57258 | 62506 | SRR29260296 |
| 1124635-R1 | Oct-21 | Processing plant 1 | present | | | 342 | 57291 | 62552 | SRR29260295 |
| 1124636-R1 | Oct-21 | Processing plant 1 | present | | | 342 | 57292 | 62553 | SRR29260294 |
| 1133625-R1 | Nov-21 | Processing plant 2 | present | | | 342 | 57297 | 62562 | SRR29260293 |
| 1157334-R1 | Jan-22 | Processing plant 1 | present | | | 342 | 25646 | 62623 | SRR29260292 |
| 1043253-R1 | Apr-21 | Processing plant 1 | present | | | 342 | 57464 | 62778 | SRR29260291 |
| 1138925-R1 | Dec-21 | Processing plant 2 | present | | | 342 | 57309 | 62575 | SRR29260516 |
| 1106709-R1 | Sep-21 | Processing plant 3 | present | | | 7790 | 57280 | 62533 | SRR29260514 |
| 1106711-R1 | Sep-21 | Processing plant 3 | present | | | 7790 | 57280 | 62534 | SRR29260513 |
| 1058017-R1 | May-21 | Processing plant 1 | present | present | | 81 | 52452 | 57530 | SRR29260512 |
| 1200021-R1 | May-22 | Processing plant 1 | present | | | 81 | 57358 | 62637 | SRR29260511 |
| 1202471-R1 | May-22 | Processing plant 1 | present | | | 81 | 57358 | 62637 | SRR29260510 |
| 1202492-R1 | May-22 | Processing plant 1 | present | | | 81 | 57358 | 62637 | SRR29260509 |
| 1200016-R1 | May-22 | Processing plant 1 | present | | | 81 | 57358 | 62637 | SRR29260508 |
| 1219865-R1 | Jul-22 | Processing plant 1 | present | | | 9246 | 57319 | 62655 | SRR29260507 |
| 1185421-R1 | Apr-22 | Processing plant 2 | present | | | 9246 | 57385 | 62672 | SRR29260506 |
| 1197053-R1 | May-22 | Processing plant 2 | present | | | 9246 | 57391 | 62682 | SRR29260505 |
| 1200015-R1 | May-22 | Processing plant 1 | present | | | 81 | 57395 | 62687 | SRR29260502 |
| 1203151-R1 | May-22 | Processing plant 1 | present | | | 81 | 57402 | 62694 | SRR29260501 |
| 1210478-R1 | Jun-22 | Processing plant 2 | present | present | | 9246 | 57406 | 62700 | SRR29260500 |
| 1208709-R1 | Jun-22 | Processing plant 1 | present | | | 9246 | 57406 | 62700 | SRR29260499 |
| 1225475-R1 | Jul-22 | Processing plant 1 | present | | | 9246 | 57319 | 62708 | SRR29260498 |
| 1215775-R1 | Jun-22 | Processing plant 1 | present | | | 81 | 57416 | 62713 | SRR29260497 |
| 1215778-R1 | Jun-22 | Processing plant 1 | present | | | 81 | 57417 | 62714 | SRR29260496 |
| 1217244-R1 | Jun-22 | Processing plant 1 | present | | | 9246 | 57419 | 62716 | SRR29260495 |
| 1218305-R1 | Jul-22 | Processing plant 1 | present | | | 9246 | 57319 | 62718 | SRR29260494 |
| 1213972-R1 | Jun-22 | Processing plant 1 | present | | | 81 | 57412 | 62707 | SRR29260493 |
| 1214349-R1 | Jun-22 | Processing plant 1 | present | | | 81 | 57413 | 62709 | SRR29260491 |

| | | | | | | | | | |
|---|---|---|---|---|---|---|---|---|---|
| 1209787-R1 | Jun-22 | Processing plant 2 | present | | | 81 | 57512 | 62839 | SRR29260490 |
| 1244892-R1 | Sep-22 | Processing plant 1 | present | | | 19009 | 57458 | 62771 | SRR29260489 |
| 1209786-R1 | Jun-22 | Processing plant 2 | present | | | 81 | 57407 | 62701 | SRR29260488 |
| 1034126-R1 | Mar-21 | Processing plant 2 | present | present | | 85 | 57462 | 62776 | SRR29260487 |
| 1153728-R1 | Jan-22 | Processing plant 1 | present | | | 80 | 25661 | 27875 | SRR29260486 |
| 1153727-R1 | Jan-22 | Processing plant 1 | present | | | 80 | 25661 | 27875 | SRR29260485 |
| 1146311-R1 | Dec-21 | Processing plant 2 | present | | present | 80 | 25661 | 60296 | SRR29260290 |
| 1153729-R1 | Jan-22 | Processing plant 1 | present | | present | 80 | 25661 | 60296 | SRR29260289 |
| 1157585-R1 | Jan-22 | Processing plant 2 | present | | | 80 | 57348 | 62625 | SRR29260288 |
| 1145621-R1 | Dec-21 | Processing plant 2 | present | | present | 18989 | 57321 | 62590 | SRR29260286 |
| 1109461-R1 | Sep-21 | Processing plant 1 | present | | | 80 | 25658 | 27872 | SRR29260285 |
| 1146307-R1 | Dec-21 | Processing plant 2 | present | | | 89 | 57324 | 62594 | SRR29260284 |
| 1146658-R1 | Dec-21 | Processing plant 2 | present | | | 89 | 57328 | 62598 | SRR29260283 |
| 1147168-R1 | Dec-21 | Processing plant 2 | present | | | 89 | 25682 | 62601 | SRR29260282 |
| 1148708-R1 | Dec-21 | Processing plant 1 | present | | | 89 | 57334 | 62606 | SRR29260281 |
| 1146306-R1 | Dec-21 | Processing plant 2 | present | | | 89 | 57481 | 62798 | SRR29260280 |
| 1148706-R1 | Dec-21 | Processing plant 1 | present | | | 89 | 25682 | 27899 | SRR29260279 |
| 1148707-R1 | Dec-21 | Processing plant 1 | present | | | 89 | 25682 | 27899 | SRR29260278 |
| 1157584-R1 | Jan-22 | Processing plant 2 | present | | | 89 | 25682 | 27899 | SRR29260277 |
| 1141367-R1 | Dec-21 | Processing plant 2 | present | | | 89 | 25682 | 27899 | SRR29260275 |
| 1209907-R1 | Jun-22 | Processing plant 3 | present | | | 7595 | 57408 | 62702 | SRR29260274 |
| 1201031-R1 | May-22 | Processing plant 3 | present | present | | 7601 | 57398 | 62690 | SRR29260273 |
| 1203029-R1 | May-22 | Processing plant 3 | present | | | 7601 | 57401 | 62693 | SRR29260272 |
| 1203027-R1 | May-22 | Processing plant 3 | present | | | 7601 | 57400 | 62692 | SRR29260271 |
| 1009110-R1 | Jan-21 | Processing plant 1 | present | present | | 62 | 57230 | 62471 | SRR29260270 |
| 1009111-R1 | Jan-21 | Processing plant 1 | present | present | | 62 | 57230 | 62471 | SRR29260269 |
| 1012370-R1 | Jan-21 | Processing plant 1 | present | present | | 62 | 57232 | 62473 | SRR29260268 |
| 1100337-R1 | Aug-21 | Processing plant 1 | present | present | | 62 | 57270 | 62521 | SRR29260267 |
| 1235759-R1 | Aug-22 | Processing plant 1 | present | present | | 18439 | 57437 | 62744 | SRR29260266 |
| 1072708-R1 | Jun-21 | Processing plant 3 | present | | | 18978 | 57244 | 62488 | SRR29260264 |
| 1080314-R1 | Jul-21 | Processing plant 3 | present | | | 5357 | 25643 | 27871 | SRR29260263 |
| 1074629-R1 | Jun-21 | Processing plant 1 | present | | | 5357 | 25643 | 27854 | SRR29260262 |
| 1074629-R2 | Jun-21 | Processing plant 1 | present | | | 5357 | 25643 | 27854 | SRR29260261 |
| 1072087-R1 | Jun-21 | Processing plant 2 | present | | | 5357 | 25708 | 27931 | SRR29260260 |
| 1072086-R1 | Jun-21 | Processing plant 2 | present | | | 5357 | 25708 | 27931 | SRR29260259 |
| 1072083-R1 | Jun-21 | Processing plant 2 | present | | | 5357 | 25708 | 27931 | SRR29260484 |
| 1071604-R1 | Jun-21 | Processing plant 2 | present | | | 5357 | 57242 | 62486 | SRR29260483 |
| 1072476-R1 | Jun-21 | Processing plant 1 | present | | | 5357 | 57243 | 62487 | SRR29260482 |
| 1074626-R1 | Jun-21 | Processing plant 1 | present | | | 5357 | 25643 | 62490 | SRR29260481 |
| 1074628-R1 | Jun-21 | Processing plant 1 | present | | | 5357 | 25643 | 62491 | SRR29260479 |
| 1076702-R1 | Jul-21 | Processing plant 1 | present | | | 5357 | 57249 | 62496 | SRR29260478 |
| 1077095-R1 | Jul-21 | Processing plant 2 | present | | | 5357 | 57250 | 62497 | SRR29260477 |
| 1082448-R1 | Jul-21 | Processing plant 1 | present | | | 5357 | 57253 | 62501 | SRR29260476 |
| 1076701-R1 | Jul-21 | Processing plant 1 | present | | | 5357 | 57249 | 62781 | SRR29260475 |
| 1085042-R1 | Jul-21 | Processing plant 2 | present | | | 5357 | 55257 | 62782 | SRR29260474 |
| 1138142-R1 | Nov-21 | Processing plant 2 | present | | | 5357 | 57305 | 62571 | SRR29260473 |
| 1074627-R1 | Jun-21 | Processing plant 1 | present | | | 5357 | 57466 | 62780 | SRR29260472 |
| 1083228-R1 | Jul-21 | Processing plant 3 | present | | | 9240 | 57255 | 62503 | SRR29260471 |
| 1103035-R1 | Sep-21 | Processing plant 2 | present | | present | 7637 | 25660 | 27874 | SRR29260470 |
| 1103041-R1 | Sep-21 | Processing plant 2 | present | | present | 7637 | 25660 | 27874 | SRR29260468 |
| 1096958-R1 | Aug-21 | Processing plant 2 | present | | | 9241 | 25670 | 27886 | SRR29260467 |
| 1191626-R1 | Apr-22 | Processing plant 2 | present | | | 9241 | 25701 | 27924 | SRR29260466 |
| 1103036-R1 | Sep-21 | Processing plant 2 | present | | | 9241 | 25670 | 27886 | SRR29260465 |
| 1234836-R1 | Aug-22 | Processing plant 2 | present | | | 9241 | 25670 | 27886 | SRR29260464 |
| 1130726-R1 | Nov-21 | Processing plant 2 | present | | | 9241 | 25670 | 27886 | SRR29260463 |
| 1100431-R1 | Aug-21 | Processing plant 2 | present | | | 9241 | 25662 | 27876 | SRR29260462 |
| 1118160-R1 | Oct-21 | Processing plant 2 | present | | | 9241 | 25670 | 27886 | SRR29260461 |
| 1100431-R2 | Aug-21 | Processing plant 2 | present | | | 9241 | 25662 | 27876 | SRR29260460 |
| 1100436-R1 | Aug-21 | Processing plant 2 | present | present | | 9241 | 25662 | 27876 | SRR29260459 |

| | | | | | | | | |
|---|---|---|---|---|---|---|---|---|
| 1110970-R1 | Sep-21 | Processing plant 2 | present | | 9241 | 25662 | 27876 | SRR29260457 |
| 1134447-R1 | Nov-21 | Processing plant 2 | present | | 9241 | 55268 | 60280 | SRR29260456 |
| 1140329-R1 | Dec-21 | Processing plant 2 | present | | 9241 | 55310 | 60339 | SRR29260455 |
| 1101970-R1 | Sep-21 | Processing plant 2 | present | | 9241 | 57276 | 62528 | SRR29260454 |
| 1110356-R1 | Sep-21 | Processing plant 2 | present | | 9241 | 57285 | 62541 | SRR29260453 |
| 1185414-R1 | Apr-22 | Processing plant 2 | present | | 9241 | 57384 | 62671 | SRR29260226 |
| 1192034-R1 | Apr-22 | Processing plant 2 | present | | 9241 | 57384 | 62678 | SRR29260225 |
| 1216323-R1 | Jun-22 | Processing plant 2 | present | | 9241 | 57410 | 62705 | SRR29260224 |
| 1211940-R1 | Jun-22 | Processing plant 2 | present | | 9241 | 57410 | 62705 | SRR29260223 |
| 1222560-R1 | Jul-22 | Processing plant 1 | present | | 19006 | 57427 | 62725 | SRR29260222 |
| 1223048-R1 | Jul-22 | Processing plant 1 | present | | 9241 | 25670 | 62726 | SRR29260220 |
| 1223050-R1 | Jul-22 | Processing plant 1 | present | | 9241 | 25670 | 62727 | SRR29260219 |
| 1224613-R1 | Jul-22 | Processing plant 2 | present | | 9241 | 57428 | 62728 | SRR29260218 |
| 1226603-R1 | Jul-22 | Processing plant 1 | present | | 9241 | 57430 | 62730 | SRR29260217 |
| 1100433-R1 | Aug-21 | Processing plant 2 | present | | 9241 | 25670 | 62783 | SRR29260606 |
| 1129164-R1 | Nov-21 | Processing plant 2 | present | | 9241 | 57472 | 62789 | SRR29260605 |
| 1223051-R1 | Jul-22 | Processing plant 1 | present | | 9241 | 57518 | 62845 | SRR29260604 |
| 1192508-R1 | Apr-22 | Processing plant 2 | present | | 9241 | 25699 | 27922 | SRR29260603 |
| 1185412-R1 | Apr-22 | Processing plant 2 | present | | 9241 | 25699 | 27922 | SRR29260602 |
| 1255201-R1 | Oct-22 | Processing plant 1 | present | | 9241 | 57357 | 62636 | SRR29260601 |
| 1283048-R1 | Dec-22 | Processing plant 1 | present | | 9241 | 57357 | 62636 | SRR29260599 |
| 1187975-R1 | Apr-22 | Processing plant 2 | present | | 9241 | 25699 | 62673 | SRR29260598 |
| 1263265-R1 | Oct-22 | Processing plant 1 | present | | 9241 | 57357 | 62821 | SRR29260597 |
| 1226220-R1 | Jul-22 | Processing plant 2 | present | | 9241 | 57519 | 62847 | SRR29260596 |
| 1267275-R1 | Nov-22 | Processing plant 2 | present | | 9241 | 57530 | 62864 | SRR29260595 |
| 1284604-R1 | Dec-22 | Processing plant 1 | present | | 17590 | 52468 | 57547 | SRR29260594 |
| 1280305-R1 | Dec-22 | Processing plant 1 | present | | 17590 | 52468 | 57547 | SRR29260593 |
| 1232535-R1 | Aug-22 | Processing plant 2 | present | present | 17589 | 52461 | 57540 | SRR29260592 |
| 1232536-R1 | Aug-22 | Processing plant 2 | present | present | 17589 | 52461 | 57540 | SRR29260591 |
| 1228734-R1 | Jul-22 | Processing plant 2 | present | present | 50 | 52463 | 57542 | SRR29260590 |
| 1230624-R1 | Aug-22 | Processing plant 2 | present | | 50 | 52463 | 57542 | SRR29260587 |
| 1230616-R1 | Aug-22 | Processing plant 1 | present | | 50 | 52463 | 57542 | SRR29260586 |
| 1233106-R1 | Aug-22 | Processing plant 2 | present | | 50 | 52463 | 57542 | SRR29260585 |
| 1233418-R1 | Aug-22 | Processing plant 1 | present | | 50 | 52463 | 57542 | SRR29260388 |
| 1234002-R1 | Aug-22 | Processing plant 1 | present | | 50 | 52463 | 57542 | SRR29260387 |
| 1236901-R1 | Aug-22 | Processing plant 2 | present | | 50 | 52463 | 57542 | SRR29260386 |
| 1234844-R1 | Aug-22 | Processing plant 2 | present | | 50 | 52463 | 57542 | SRR29260385 |
| 1231913-R1 | Aug-22 | Processing plant 2 | present | | 50 | 52463 | 57542 | SRR29260384 |
| 1236895-R1 | Aug-22 | Processing plant 2 | present | | 50 | 52463 | 57542 | SRR29260383 |
| 1241499-R1 | Sep-22 | Processing plant 1 | present | | 50 | 52463 | 57542 | SRR29260382 |
| 1242951-R1 | Sep-22 | Processing plant 2 | present | | 50 | 52463 | 57542 | SRR29260380 |
| 1241500-R1 | Aug-22 | Processing plant 1 | present | | 50 | 52463 | 57542 | SRR29260379 |
| 1240596-R1 | Aug-22 | Processing plant 2 | present | | 50 | 52463 | 57542 | SRR29260378 |
| 1240730-R1 | Aug-22 | Processing plant 2 | present | | 50 | 52463 | 57542 | SRR29260377 |
| 1244493-R1 | Sep-22 | Processing plant 1 | present | | 50 | 52463 | 57542 | SRR29260376 |
| 1232532-R1 | Aug-22 | Processing plant 2 | present | | 50 | 52463 | 62735 | SRR29260375 |
| 1233420-R1 | Aug-22 | Processing plant 1 | present | | 50 | 52463 | 62737 | SRR29260374 |
| 1234001-R1 | Aug-22 | Processing plant 1 | present | | 50 | 52463 | 62740 | SRR29260373 |
| 1242944-R1 | Sep-22 | Processing plant 2 | present | | 50 | 57452 | 62764 | SRR29260372 |
| 1242947-R1 | Sep-22 | Processing plant 2 | present | | 50 | 57454 | 62766 | SRR29260371 |
| 1242955-R1 | Sep-22 | Processing plant 1 | present | | 50 | 57455 | 62767 | SRR29260369 |
| 1243354-R1 | Sep-22 | Processing plant 1 | present | | 50 | 57457 | 62769 | SRR29260368 |
| 1243357-R1 | Sep-22 | Processing plant 2 | present | | 50 | 52463 | 62770 | SRR29260367 |
| 1250717-R1 | Sep-22 | Processing plant 1 | present | | 50 | 57460 | 62774 | SRR29260366 |
| 1254633-R1 | Oct-22 | Processing plant 2 | present | | 50 | 57460 | 62774 | SRR29260365 |
| 1215331-R1 | Jun-22 | Processing plant 1 | present | | 50 | 57515 | 62842 | SRR29260364 |
| 1233107-R1 | Aug-22 | Processing plant 2 | present | present | 50 | 52463 | 62851 | SRR29260363 |
| 1236893-R1 | Aug-22 | Processing plant 1 | present | | 50 | 52463 | 62854 | SRR29260362 |
| 1242946-R1 | Sep-22 | Processing plant 2 | present | | 19015 | 57525 | 62858 | SRR29260361 |

| | | | | | | | | | | |
|---|---|---|---|---|---|---|---|---|---|---|
| 1250530-R1 | Sep-22 | Processing plant 2 | present | | | | 50 | 57527 | 62860 | SRR29260360 |
| 1242654-R1 | Sep-22 | Processing plant 2 | present | | | | 50 | 52463 | 62759 | SRR29260358 |
| 1215336-R1 | Jun-22 | Processing plant 1 | present | | | | 50 | 57414 | 62711 | SRR29260357 |
| 1234170-R1 | Aug-22 | Processing plant 2 | present | | | | 50 | 57435 | 62741 | SRR29260452 |
| 1097768-R1 | Aug-21 | Processing plant 3 | present | | | | 49 | 57268 | 62518 | SRR29260451 |
| 1211123-R1 | Jun-22 | Processing plant 2 | present | | | | 19001 | 57397 | 62689 | SRR29260450 |
| 1210475-R1 | Jun-22 | Processing plant 2 | present | | | | 19001 | 57397 | 62689 | SRR29260449 |
| 1211112-R1 | Jun-22 | Processing plant 2 | present | | | | 19001 | 57397 | 62703 | SRR29260448 |
| 1213172-R1 | Jun-22 | Processing plant 2 | present | | | | 19001 | 57411 | 62706 | SRR29260447 |
| 1213251-R1 | Jun-22 | Processing plant 2 | present | | | | 19001 | 57514 | 62841 | SRR29260446 |
| 1215773-R1 | Jun-22 | Processing plant 1 | present | | | | 81 | 57415 | 62712 | SRR29260445 |
| 1241498-R1 | Aug-22 | Processing plant 1 | present | | | | 19007 | 57447 | 62757 | SRR29260443 |
| 1242945-R1 | Sep-22 | Processing plant 2 | present | | | | 19008 | 57453 | 62765 | SRR29260442 |
| 1247373-R1 | Sep-22 | Processing plant 1 | present | | | | 50 | 57459 | 62772 | SRR29260441 |
| 1138085-R1 | Nov-21 | Processing plant 3 | present | | | | 19010 | 57474 | 62791 | SRR29260440 |
| 1281077-R1 | Dec-22 | Processing plant 1 | present | | present | present | 46 | 55325 | 60356 | SRR29260439 |
| 1281534-R1 | Dec-22 | Processing plant 1 | present | | | present | 46 | 55326 | 60357 | SRR29260438 |
| 1071548-R1 | Jun-21 | Processing plant 1 | present | | present | | 62 | 55261 | 60269 | SRR29260437 |
| 1282140-R1 | Dec-22 | Processing plant 1 | present | | present | | 18447 | 55328 | 60359 | SRR29260436 |
| 1113816-R1 | Oct-21 | Processing plant 2 | present | | present | | 342 | 25648 | 60320 | SRR29260435 |
| 1151548-R1 | Jan-22 | Processing plant 2 | present | | | | 342 | 52455 | 57534 | SRR29260434 |
| 1118148-R1 | Oct-21 | Processing plant 1 | present | | | | 342 | 55277 | 60295 | SRR29260432 |
| 1118149-R1 | Oct-21 | Processing plant 1 | present | | | | 342 | 55277 | 60295 | SRR29260431 |
| 1067192-R1 | Jun-21 | Processing plant 1 | present | | | | 81 | 25654 | 27867 | SRR29260430 |
| 1067193-R1 | Jun-21 | Processing plant 1 | present | | | | 81 | 25654 | 27867 | SRR29260429 |
| 1069546-R1 | Jun-21 | Processing plant 1 | present | | | | 81 | 25654 | 27867 | SRR29260428 |
| 1069542-R1 | Jun-21 | Processing plant 1 | present | | | | 81 | 25654 | 27867 | SRR29260427 |
| 1067722-R1 | Jun-21 | Processing plant 1 | present | | | present | 81 | 25654 | 27867 | SRR29260426 |
| 1069544-R1 | Jun-21 | Processing plant 1 | present | | | | 81 | 25654 | 27867 | SRR29260425 |
| 1069545-R1 | Jun-21 | Processing plant 1 | present | | | | 81 | 25654 | 27867 | SRR29260424 |
| 1065550-R1 | Jun-21 | Processing plant 2 | present | | | | 81 | 52452 | 57530 | SRR29260423 |
| 1069541-R1 | Jun-21 | Processing plant 1 | present | | | | 81 | 55259 | 60267 | SRR29260421 |
| 1151687-R1 | Jan-22 | Processing plant 1 | present | | | | 80 | 25661 | 27875 | SRR29260356 |
| 1151688-R1 | Jan-22 | Processing plant 1 | present | | | | 80 | 25661 | 27875 | SRR29260355 |
| 1116660-R1 | Oct-21 | Processing plant 2 | present | | | | 80 | 25661 | 60289 | SRR29260354 |
| 1151651-R1 | Jan-22 | Processing plant 1 | present | | | present | 80 | 55297 | 60325 | SRR29260353 |
| 1148790-R1 | Dec-21 | Processing plant 2 | present | | | | 89 | 25682 | 27899 | SRR29260352 |
| 1150155-R1 | Jan-22 | Processing plant 2 | present | | | | 18442 | 55287 | 60308 | SRR29260351 |
| 1066488-R1 | Jun-21 | Processing plant 1 | present | | present | | 62 | 55254 | 60262 | SRR29260350 |
| 1115898-R1 | Oct-21 | Processing plant 1 | present | | present | | 18439 | 55260 | 60285 | SRR29260349 |
| 1115899-R1 | Oct-21 | Processing plant 1 | present | | present | | 18439 | 55260 | 60286 | SRR29260348 |
| 1115900-R1 | Oct-21 | Processing plant 1 | present | | present | | 18439 | 55260 | 60287 | SRR29260346 |
| 1115911-R1 | Oct-21 | Processing plant 1 | present | | present | | 18439 | 55271 | 60288 | SRR29260345 |
| 1152332-R1 | Jan-22 | Processing plant 1 | present | | present | | 18439 | 55260 | 60303 | SRR29260344 |
| 1152335-R1 | Jan-22 | Processing plant 1 | present | | present | | 18439 | 55260 | 60303 | SRR29260343 |
| 1152334-R1 | Jan-22 | Processing plant 1 | present | | present | | 18439 | 55260 | 60303 | SRR29260342 |
| 1149209-R1 | Dec-21 | Processing plant 1 | present | | present | | 18439 | 55260 | 60303 | SRR29260341 |
| 1118163-R1 | Oct-21 | Processing plant 1 | present | | present | | 18439 | 55295 | 60323 | SRR29260340 |
| 1152333-R1 | Jan-22 | Processing plant 1 | present | | present | | 18439 | 55301 | 60329 | SRR29260339 |
| 1150858-R1 | Jan-22 | Processing plant 1 | present | | present | | 18439 | 55291 | 60315 | SRR29260338 |
| 1114579-R1 | Oct-21 | Processing plant 1 | present | | present | | 18439 | 55260 | 60283 | SRR29260337 |
| 1066707-R1 | Jun-21 | Processing plant 2 | present | | | | 5357 | 25643 | 27854 | SRR29260335 |
| 1070449-R1 | Jun-21 | Processing plant 2 | present | | | | 5357 | 25643 | 27854 | SRR29260334 |
| 1115233-R1 | Oct-21 | Processing plant 2 | present | | | | 9241 | 25670 | 27886 | SRR29260333 |
| 1282043-R1 | Dec-22 | Processing plant 2 | present | | | | 17590 | 52468 | 57547 | SRR29260332 |
| 1280992-R1 | Dec-22 | Processing plant 2 | present | | | | 17590 | 52468 | 57547 | SRR29260331 |
| 1281535-R1 | Dec-22 | Processing plant 1 | present | | | | 17590 | 52468 | 57547 | SRR29260330 |
| 1152501-R1 | Jan-22 | Processing plant 1 | present | | | | 9243 | 25677 | 27893 | SRR29260329 |
| 1236512-R1 | Aug-22 | Processing plant 2 | present | | | present | 50 | 55313 | 60344 | SRR29260328 |

| | | | | | | | | |
|---|---|---|---|---|---|---|---|---|
| 1236879-R1 | Aug-22 | Processing plant 2 | present | | 50 | 55314 | 60345 | SRR29260327 |
| 1282139-R1 | Dec-22 | Processing plant 1 | present | present | 18447 | 55327 | 60358 | SRR29260326 |

Supplementary table S3: Comparison of MGT8 and MGT9 STs with whole genome SNP types

| | **Number of STs** | | |
|---|---|---|---|
| **MGT level** | **1 SNP type: 1 MGT ST (Identical resolution)** | **1 MGT ST, > 1 SNP types: (SNP higher resolution)** | **1 SNP type: > 1 MGT STs (MGT higher resolution)** |
| **MGT9** | 192 (80.33%) | 10 (4.2%) | 37(15.48%) |
| **MGT8** | 151(75.88%) | 27(13.57%) | 21(10.55%) |

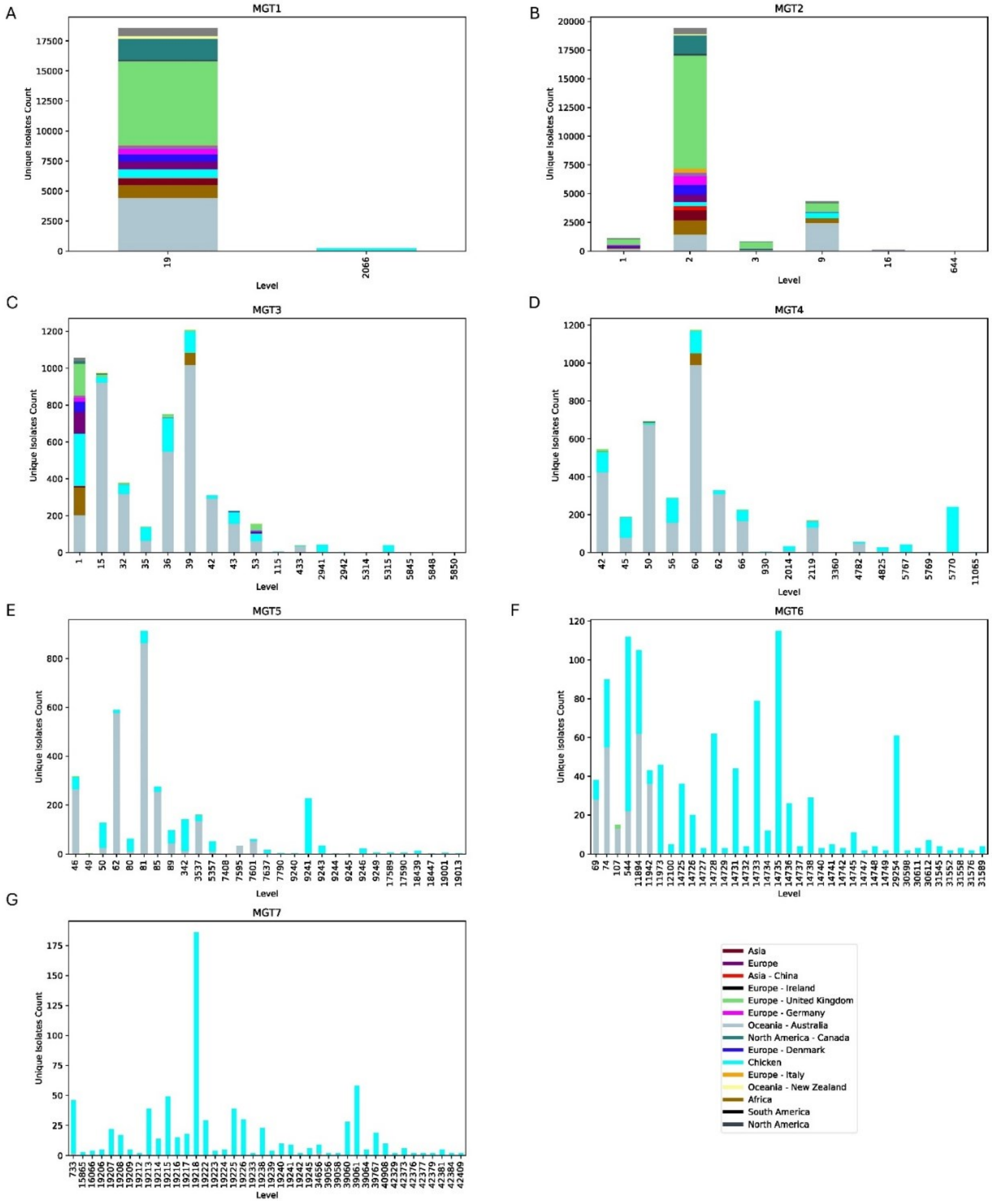


Supplementary figure S1: Comparison with chicken MGT STs and global historical human STs from MGT1-MGT7. a) Comparison of MGT1 STs of chicken STm with global human MGT1 STs. b) Comparison of MGT2 STs of chicken STm with global human MGT2 STs. c) Comparison of MGT3 STs of chicken STm with global human MGT3 STs. d) Comparison of MGT4 STs of chicken STm with global human MGT4 STs. e) Comparison of MGT5 STs of chicken STm with global human MGT5 STs. f) Comparison of MGT6 STs of chicken STm with global human MGT6 STs. g) Comparison of MGT7 STs of chicken STm with global human MGT7 STs.

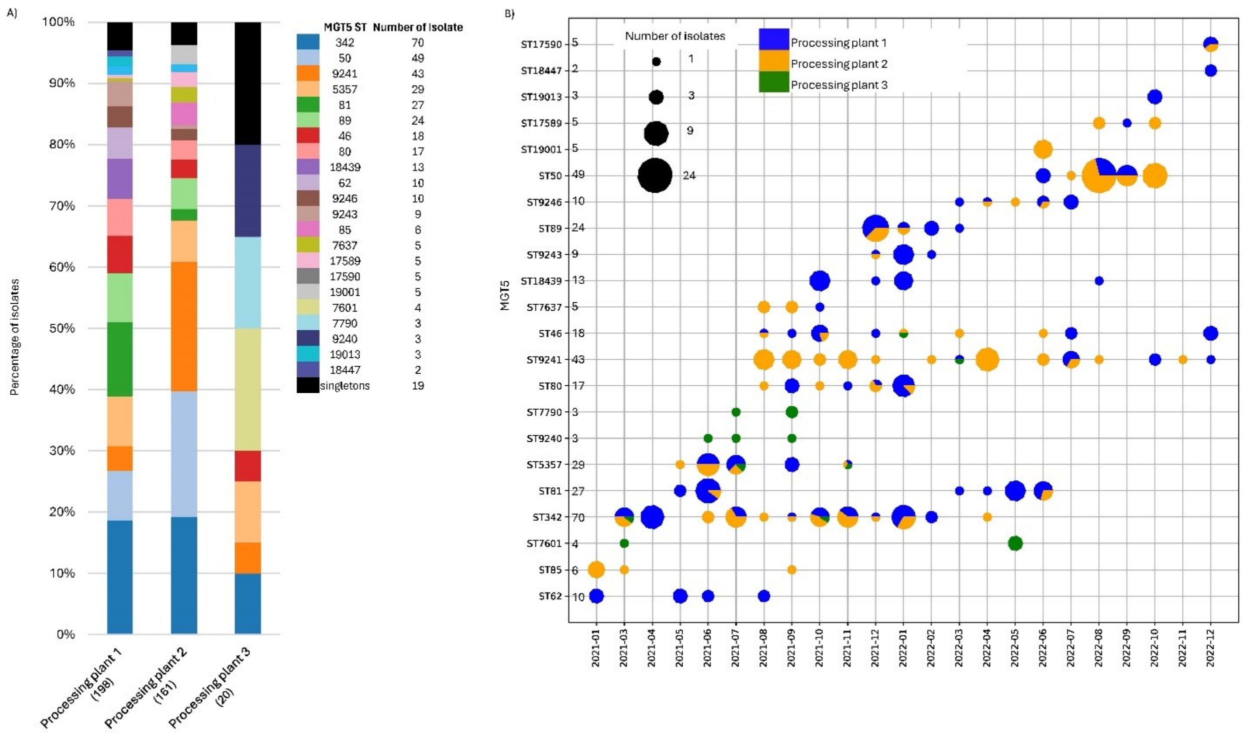

Supplementary figure S2: Temporal and spatial dynamics of isolates at MGT5 level. A) Spatial distribution of chicken STm population at the MGT5 level in the three geographically distant processing plants. The number of isolates collected from each plant was shown within brackets. The y axis is the percentage of STs isolated from each plant. The color legend identifies each MGT5 STs. B) Distribution of MGT5 STs with multiple isolates per ST across the three processing plants and temporal patterns throughout the collection period. Y axis lists STs and number of isolates while X axis marks the year and month of isolate collection. Note the discontinuity of months as months without isolates are not shown. The colors of the dots represent processing plants, and the size of the dots represents the number of isolates as shown in the legends.

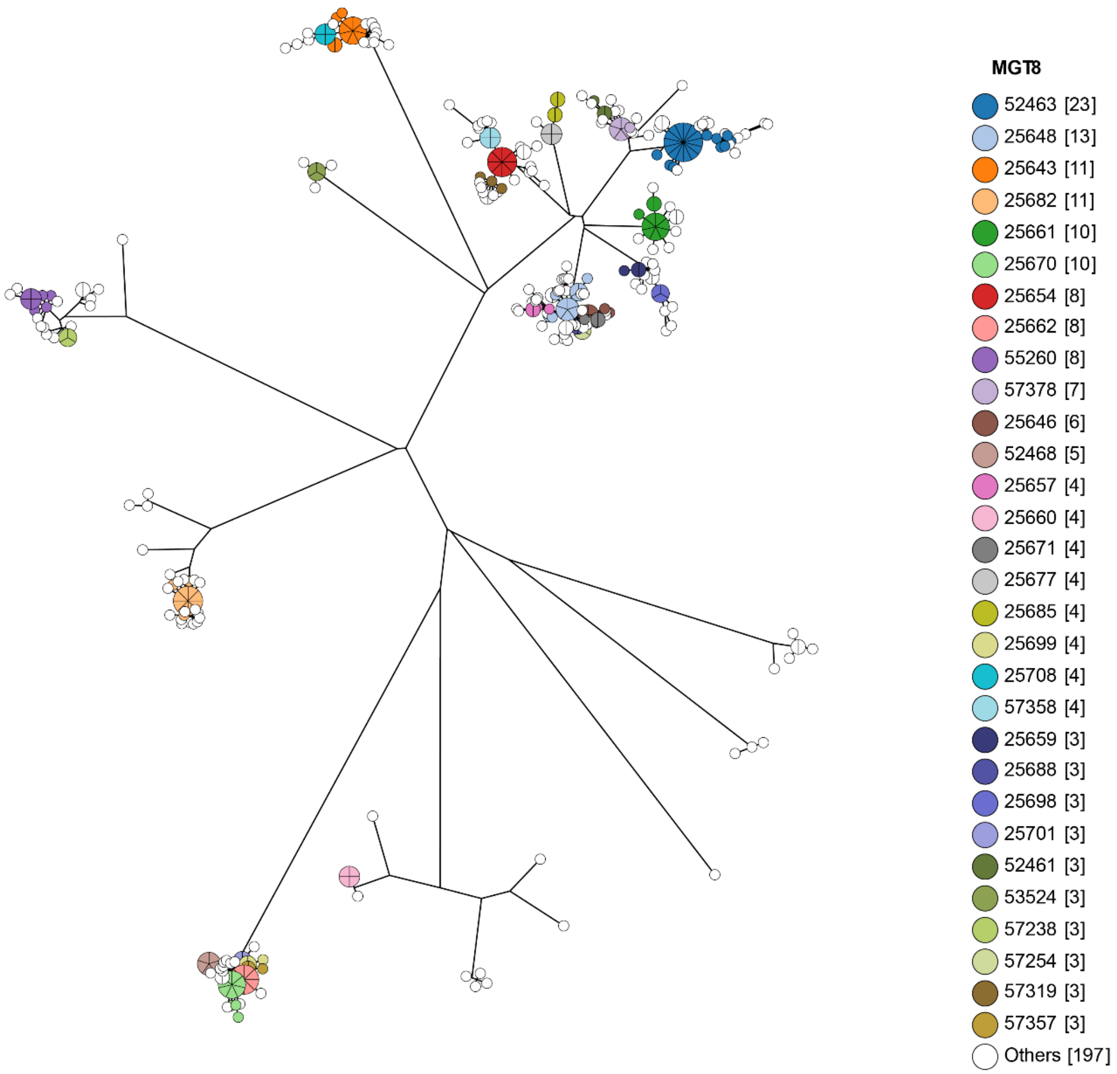


Supplementary figure S3: Population structure of chicken STm isolates at the MGT8 level. The phylogenetic tree was constructed using MGT9 allelic profiles. Each round dot at the tip of the tree represents an MGT9 ST. MGT8 STs were overlaid onto the tree per color legend to visualise MGT9 STs grouped by MGT8 STs and phylogenetic clustering of MGT5 STs. The number in brackets in the colour legend is the number of isolates of a given MGT8 ST.

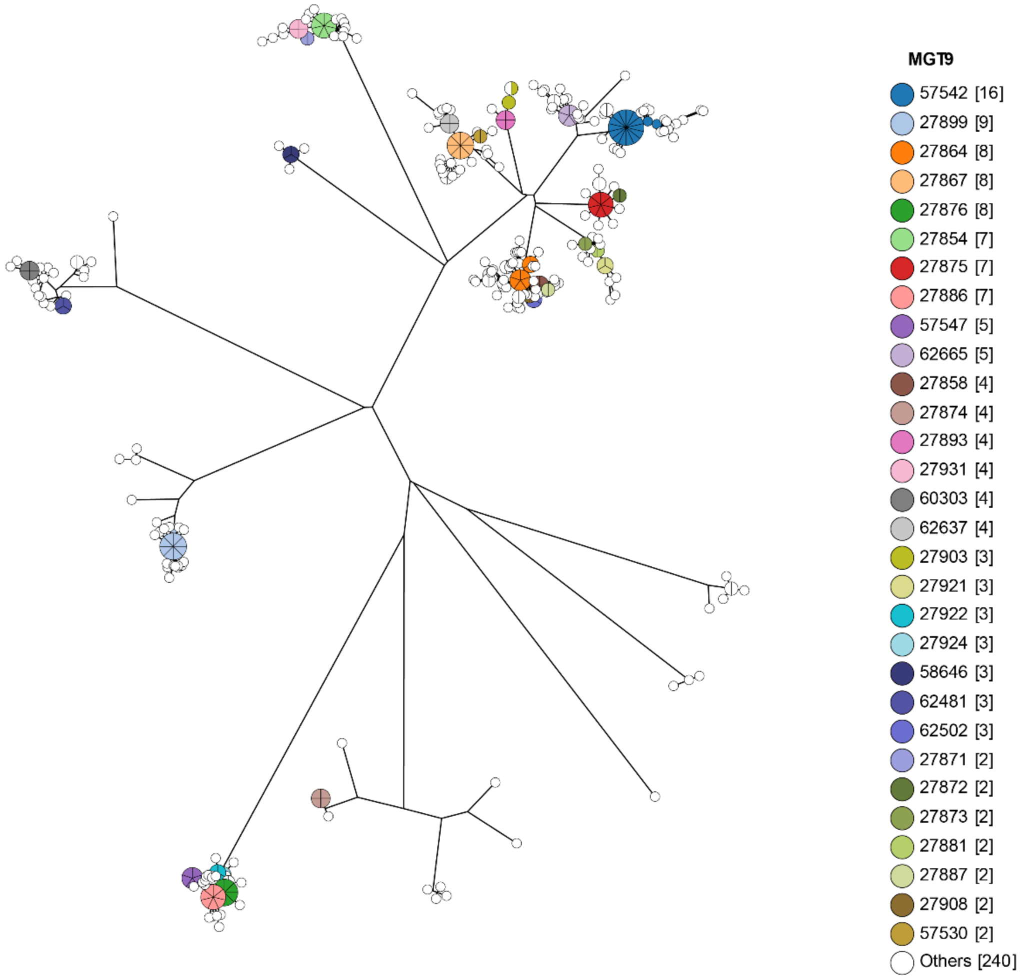


Supplementary figure S4: Population structure of chicken STm isolates at the MGT9 level. The phylogenetic tree was constructed using MGT9 allelic profiles. Each round dot at the tip of the tree represents an MGT9 ST. The number in brackets in the colour legend is the number of isolates of a given MGT9 ST.
